# Promoting Mental Health and Psychological Thriving in University Students: A Randomized Controlled Trial of Three Well-Being Interventions

**DOI:** 10.3389/fpsyt.2020.00590

**Published:** 2020-07-15

**Authors:** Emma M. Seppälä, Christina Bradley, Julia Moeller, Leilah Harouni, Dhruv Nandamudi, Marc A. Brackett

**Affiliations:** ^1^ Yale Child Study Center & Yale Center for Emotional Intelligence, Yale University, New Haven, CT, United States; ^2^ Center for Compassion and Altruism Research and Education, Stanford University, Stanford, CA, United States; ^3^ Department of Education, Leipzig University, Leipzig, Germany; ^4^ Yale School of Management, Yale University, New Haven, CT, United States; ^5^ Medical Research Council Cognition and Brain Sciences Unit, University of Cambridge, Cambridge, United Kingdom

**Keywords:** emotional intelligence, mindfulness-based stress reduction, Sudarshan Kriya, college mental health, well-being, sky campus happiness, depression, anxiety

## Abstract

This study aimed to address the decline in mental health on U.S. university campuses by examining the effects of three interventions. University students suffer from high levels of anxiety, depression, and suicide. Counseling centers on university campuses are struggling to meet increased demand. The cost to students and universities could be buffered by offering preventative, psychoeducational, and skill-building training programs that promote mental health and psychological thriving. To date, the research literature has not yielded systematically evaluated and recommendable preventative mental health and well-being programs for university students. In a registered, randomized controlled trial, 131 university students were either placed in a non-intervention control group (*N* = 47) or received training in one of three 30-hour, eight-week semester-long well-being programs: SKY Campus Happiness (“SKY”; *N* = 29), Foundations of Emotional Intelligence (“EI”; *N* = 21) or Mindfulness-Based Stress Reduction (“MBSR”; *N* = 34). Compared to the control group and controlling for variance of baseline measurements and multiple comparisons, SKY Campus Happiness showed the greatest impact, benefiting six outcomes: depression, stress, mental health, mindfulness, positive affect and social connectedness. EI benefited one outcome: mindfulness. The MBSR group showed no change. Delivering SKY or EI to university students may be a cost-effective and efficient way to proactively and preventatively address mental health for university students and reduce the financial strain on universities.

## Introduction

The World Health Organization defines health as “a state of complete physical, mental and social well-being and not merely the absence of disease or infirmity” (1). Similarly, we can think of mental health as not merely the absence of mental illness (e.g., anxiety and depression), but also the presence of psychological thriving (e.g., gratitude, social connectedness, mindfulness). 

Mental health in college and university students in the United States has declined over the last decade (2). College-aged adults (18–25 years of age) are at greatest risk for mental illness (3) and have the highest prevalence of any type of mental illness [25.8%; (4)]. The most common self-reported concerns by university students visiting counseling centers in the United States between 2017 and 2018 were anxiety (58.9%), depression (48.0%) and stress (46.9%) (5, 6). A 2018 American College Health Association survey of 88,178 college students across 140 campuses reported that 60% of students self-reported experiencing overwhelming anxiety and 40% self-reported feeling so depressed they had difficulty functioning (7). 

Mental illness interferes with student learning and is significantly associated with lower GPA (7, 8). Mental illness is also associated with substance abuse in university students in the United States (9), which further negatively impacts academic performance (10) and exacerbates mental illness. 

Mental illness and substance abuse are associated with more than 90% of all cases of suicide in the United States (11, 12). While suicide is the tenth leading cause of death in the United States (13), it is the second leading cause of death for college-aged students, after traffic accidents (14). Recent years have seen a record increase in suicides by young adults aged 15–24 years old (13). The highest number of suicides ever reported for this age group was in 2017 (2, 13, 15). From 2000 to 2016, over 10% of students reported having seriously considered suicide in the past year (2, 5–7, 11, 13, 16). 

Whereas social connection, a measure of psychological thriving, is predictive of emotional health, mental illness is often associated with loneliness and isolation which in turn can further deteriorate mental health and increase suicidal ideation (17–24). 

Campus counseling centers are inadequately prepared to meet increased demand for services. From 2009 to 2014, campus counseling centers experienced an average increase of 30% of students seeking treatment for an average 6% institutional enrollment increase (25). Ninety-four percent of counseling center directors report an increasing number of students with severe diagnosed mental health problems presenting on their campuses (26), and 57% of directors indicate that their resources are insufficient to meet students’ needs (5). 

The traditional approach to addressing mental illness is to address symptoms after they have presented themselves. Students are typically diagnosed and then prescribed medication, counseling or a combination thereof (27, 28). A steady increase in demand for counseling, however, makes this recourse financially unsustainable (29). Moreover, medications often present with aversive side effects (4) that can disrupt cognitive functions [e.g., attention and memory, essential for succeeding in college (30)]. Finally, these types of interventions are not only applied post-hoc but also focus exclusively on treating mental illness symptoms without setting up the conditions for psychological resilience and thriving.

A preventative and proactive approach to mental health that provides students with empirically validated tools for psychological resilience and thriving *prior* to developing symptoms may be a viable additional support to the efforts made by campus counseling centers. Several studies indicate that skill-building interventions are effective in improving the lives of adolescents and college students (31–33). A recent meta-analysis of universal mental health prevention programs on university campuses showed that the most successful programs included skill-building with supervised practice (34). These programs, as compared with skill-building programs without supervised practice or those with only psychoeducational curricula, were significantly more successful in reducing anxiety, stress, depression, and distress and improving social and emotional skills (34).

However, most mental health prevention programs offered by campuses tend to fail due to a primary focus on psychoeducation (34). Without the components of *skill-building* (including practice time, reflection, questions and discussion) and supervised practice, integration of the material is challenging (34). Overall, the research literature has not yielded systematically evaluated and recommendable skill-building protocols for psychological resilience that university administrators can implement (35). Our study aimed to address this gap in the literature: we examined the impacts of three different skill-building prevention interventions with supervised practice that past research suggests could improve student mental health.

We selected SKY Campus Happiness (SKY) because it has been shown to increase psychological resilience (36, 37), decrease stress (38) and reduce impulsive behavior (39) in students. In general populations, SKY has also been shown to improve emotion regulation (40), decrease stress (41–43), anxiety and depression (44–50), and reduce PTSD (51, 52).

The second program we selected was Foundations in Emotional Intelligence (EI), a program that was adapted from both a university course and a pre-existing evidence-based approach to social and emotional learning, RULER (53). RULER has shown promising results for school-aged children in quasi-experimental and randomized controlled studies. In particular, RULER, has been shown to increase social and emotional competence (54), improve academic grades (54), emotion skills (54), cooperation (55), social problem-solving skills (56), and student-teacher relationships (57).

The third program we selected was Koru Mindfulness. Mindfulness-based interventions have been shown to reduce psychological distress (58, 59), stress (60), and anxiety (61) in university students. In general populations it has also been shown to reduce depression (62), anxiety (62), and stress symptoms (63), and to increase self-compassion (64), self-esteem (65), sleep quality (65), and physical health (66).

Since mental health may be considered on a spectrum from mental illness to psychological thriving, we aimed to measure the impact of the interventions with measures spanning that spectrum (from anxiety and depression to gratitude and social connectedness). We hypothesized that the manner in which each workshop would impact mental health would differ depending on the focus of the intervention curricula. Based on past research, we hypothesized that all three groups would benefit measures of mental health such as anxiety, stress and depression. We also hypothesized that the interventions would improve measures of psychological thriving. Given past research, we further hypothesized that SKY would also improve psychological resilience measures. We hypothesized that EI, given its emphasis on emotion regulation, would also improve positive affect. With its focus on mindfulness and self-compassion, we hypothesized that Koru Mindfulness would also benefit mindfulness and self-compassion.

An initial pilot study compared three well-being/skill-building interventions—SKY Campus Happiness (SKY), Foundations of Emotional Intelligence (EI), and Koru Mindfulness—to an inactive control group. The majority of the 203 pilot undergraduate participants recommended the well-being interventions. However, largely no effects were observed in self-reported well-being. The lack of intervention effects despite high likability was potentially due to low exposure and practice of the techniques and strategies taught during intervention delivery (10 h over 5 weeks) and lack of at-home practice (mode days of reported at-home practice was zero).

Building on the pilot, our pre-registered main study (Clinicaltrials.gov, Registration number: NCT03229577) had the same randomized controlled design with more intervention exposure (30 h over 8 weeks) in addition to at-home practice requirements. SKY and EI expanded the curriculum to include 8 weeks of content. We used the Mindfulness-Based Stress Reduction program instead of Koru Mindfulness since its curriculum is designed for an 8-week period. The same self-reported well-being measures were used here as in the pilot.

## Materials and Methods

### Recruitment

Undergraduate students from a large, four-year private university learned about the study through departmental email lists. Applicants who had participated in programs similar to the study interventions or had significant mindfulness expertise were excluded. One hundred ninety-three students committed to joining the semester-long study.

**Figure 1:**
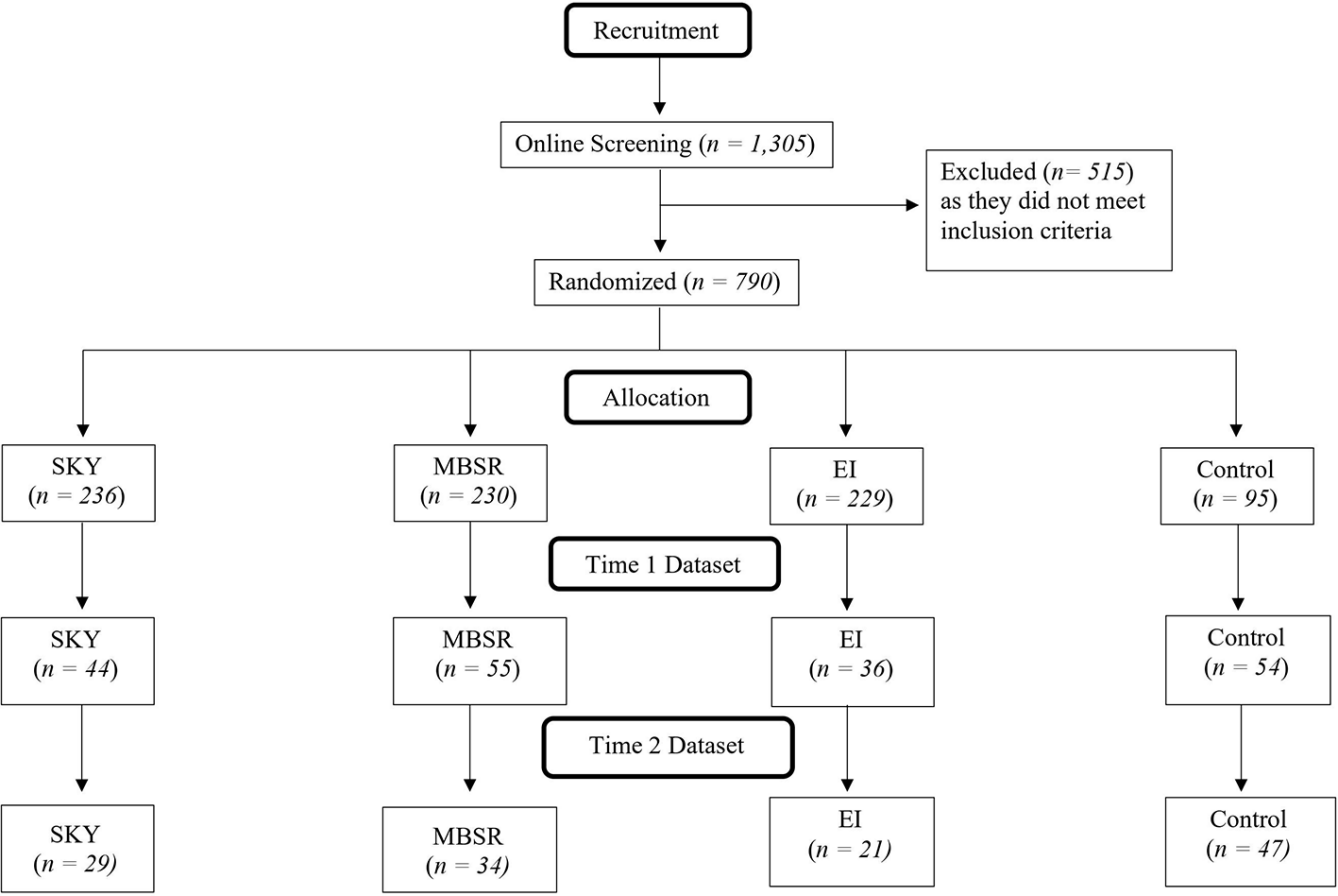
Diagram summarizing participant flow for recruitment, allocation, and analysis.

A total of 1,305 students declared an initial interest in the study. See **Figure 1** for the process by which the final participant sample was formed. Participants were excluded from this study if they had previously partaken in any of the study’s interventions or similar programs. Students considered “experts” in mindfulness (who practiced any type of mindful practice five or more times a week) were also excluded. Likewise, students who participated in the previous year’s pilot study were excluded from participation. Based on these criteria, 515 students were excluded from the study.

The remaining sample of 790 prospective students were randomly distributed into the study’s four groups: SKY, MBSR, EI, and control. After randomization, each student interested in participating in the study was provided with the schedule and requirements. Prospective participants at this time were invited to enroll, committing to pre- and post-testing, attendance and dedication requirements. Of the 790 prospective participants, 193 students accepted the invitation to join the study.

During the course of the study, fifty-eight students dropped out due to schedule conflicts (N = 11), inability to meet the time commitment (N = 9), dismissal for excessive absences (N = 4), discontinued correspondence with the researchers (N =11), physical health reasons (N = 1), mental health reasons (N = 1) or unknown reasons (N = 21). One hundred and thirty-five students completed the study. Another three participants were removed for having extreme outlier data on ten or more outcome variables. A fourth participant was removed for reporting lying on all of their practice logs.

The final sample included 131 participants with an average age of 19.67 years (*SD* = 1.02). 56.5% of participants were White, 30.5% Asian, 16.0% Hispanic or Latino, 13.0% Black or African American, 6.1% two or more races, 3.8% American Indian or Alaskan Native, 1.5% other, and 0.8% declined to provide information. Thirty-five and one-half percent of participants were male, 59.5% female, 3.1% genderqueer/gender non-conforming, and 0.8% reported “different identity”.

There were no significant differences between groups in gender, mental health diagnoses, current psychological treatment, or personality [measured by the Big Five Inventory, (67)].

Descriptive statistics for all measures at T1 and T2 can be found in Table A (overall descriptives) and Table B (group-specific descriptives) in **Supplementary Materials**.

### Procedure

Exactly one week prior to the interventions, all participants completed Time 1 (T1) questionnaires in person after providing written informed consent for the protocol approved by the Yale University Institutional Review Board. Exactly three days after the last intervention session, all participants completed the Time 2 (T2) questionnaires in person. Students practiced techniques learned in class at least three times per week and filled out practice logs. Active group participants were compensated $300. Control group participants were compensated $80.

### Interventions

The three interventions (SKY, EI and MBSR) offered equal dosage of instruction: classes were taught twice per week for a total of 30 h over eight weeks during the course of a university semester (with a two-week break in the middle for spring break). The 30 h of intervention included one or more short retreats in addition to class time. Attendance was mandatory (2 absences were allowed). Interventions were delivered by certified facilitators with seven or more years of teaching experience. Facilitators were blind to research hypotheses, data collection, and analyses.

#### SKY Campus Happiness (SKY)

SKY is a university leadership and well-being program (campushappiness.org) that includes stress-management and tools for psychological resilience: yoga postures, breathing exercises, a breath-based meditation technique [Sudarshan Kriya Yoga*;* (68)]. SKY also includes positive psychology skills (e.g., gratitude, social connection, acts of kindness, meaning and purpose). In addition, the curriculum includes discussion and application of leadership skills and service learning.

#### Foundations of Emotional Intelligence (EI)

EI is an emotional intelligence program focused on teaching knowledge of emotions and emotion regulation (54). EI was adapted from a university course on emotional intelligence and an evidence-based approach to social and emotional learning for school-aged children, RULER (www.rulerapproach.org). RULER is an acronym that stands for the five skills of emotional intelligence: recognizing, understanding, labeling, expressing and regulating emotions.

#### Mindfulness-Based Stress Reduction (MBSR)

MBSR is a mindfulness meditation program [https://www.umassmed.edu/cfm/mindfulness-based-programs/, (69)] designed for “progressive acquisition of mindful awareness, of mindfulness” (66). It involves instruction in three formal techniques: mindfulness meditation, body scanning (systematic awareness of different parts of the body from toes to the head) and simple yoga postures. In addition, the class includes discussions about meditation and its practical application to daily life.

### Measures

Outcome variables were divided along three categories: mental health, psychological thriving and health outcomes (physical health and sleep). We used a wide range of self-reported outcome measures to ensure reliable capture of change. Self-report measures assessed the effects of the programs at T1 and T2 along these categories. Participants completed two additional measures not included here: an emotion perception task and the Human Well-Being Scale. Both are in the development phase and unvalidated. In addition, participants completed a measure of creativity not related to well-being, not included here. 

#### Mental Health

##### Burnout

The Single-Item Measure of Burnout (70, 71) contains the item: “Overall, based on your definition of burnout, how would you rate your level of burnout?”. Participants choose one answer ranging from 1 = *I enjoy my work. I have no symptoms of burnout* to 5 = *I feel completely burned out and often wonder if I can go on. I am at the point where I may need some changes or may need to seek some sort of help.*

##### Stress

The Perceived Stress Scale 10-Item Inventory [PSS-10; (72); Cronbach’s Alpha=0.85] asks how frequently participants felt stress or had difficulties coping with life stresses on a scale ranging from 1 = *never* to 4 = *very often.* Example items include, “In the last month, how often have you felt that you were unable to control the important things in your life?” and “In the last month, how often have you felt that things were going your way?”.

##### Distress, Anxiety and Depression.

The Mood and Anxiety Symptom Questionnaire [MASQ-D30; (73)] consists of three subscales that measure: general distress symptoms (Cronbach’s Alpha=0.87), anhedonic depressive symptoms (Cronbach’s Alpha=0.80), and anxious arousal symptoms (Cronbach’s Alpha=0.90). Items were rated on a scale of 1 = v*ery slightly or not at all* to 5 = *extremely*. Examples of items include, “In the last two weeks I felt irritable” or “felt optimistic.”

##### Mental Health

Participants answered the question: “In general, would you say your health is…?” Items were rated on a scale 1 = *poor* to 5 = *excellent.* This self-assessment item was modeled from the Center for Disease Control’s (CDC) National Health Survey (12), and The RAND Corporation’s 36-Item Short Form Health Survey [SF-36; (74)].

#### Psychological Thriving

##### Psychological Well-Being

The Ryff Scale of Psychological Well-being is an 18-item scale [RYFF; (75)]. Items are phrased as statements about the self, to which participants rate their agreement on a scale of 1 = *strongly disagree* to 7 = *strongly agree*. Examples of items include, “I like most parts of my personality” and “The demands of everyday life often get me down.” This scale includes six subscales, each measured using three items.: Autonomy (Cronbach’s Alpha=0.70), Environmental Mastery (Cronbach’s Alpha=0.61), Personal Growth (Cronbach’s Alpha=0.48), Positive Relations (Cronbach’s Alpha=0.67), Purpose in Life (Cronbach’s Alpha=0.35), Self-acceptance (Cronbach’s Alpha=0.72).

##### Life Satisfaction

The Satisfaction with Life Scale [SWLS; (76); Cronbach’s Alpha=0.82] is a five-item scale. Items are rated on a scale from 1 = *strongly disagree* to 7 = *strongly agree*. Examples of items include, “The conditions of my life are excellent” and “If I could live my life over, I would change almost nothing.”

##### Positive Affect

The Positive and Negative Affect Schedule [PANAS; (77); Cronbach’s Alpha=0.89] is a 20-item scale containing two subscales each containing ten items: pleasant emotions and unpleasant emotions. Participants rate the extent to which they feel specific emotions over the past week on a scale from 1 = *very slightly or not at all* to 5 = *extremely*. Examples of emotions include “Excited”, “Nervous”, “Ashamed”, and “Determined”.

##### Negative Affect

The Positive and Negative Affect Schedule, see Positive Affect, above (Cronbach’s Alpha=0.87).

##### Gratitude

The Gratitude Questionnaire-Six Item Form [GQ-6; (78); Cronbach’s Alpha=0.86] is a 6-item measure. Items are rated on a scale from 1 = *strongly disagree* to 7 = *strongly agree*. Examples of items include, “I have so much in my life to be thankful for” and “Long amounts of time can go by before I feel grateful to something or someone.”

##### Self-Compassion

The Self-Compassion-Short Form [SCS-SF; (79); Cronbach’s Alpha=0.89] is a 12-item scale measuring the three components of self-compassion: self-kindness, common humanity, and mindfulness. Participants rate how often they act in the presented manner using a five-point scale from 1 = *almost never* to 5 = *almost always*. Examples of items include “I try to be understanding and patient toward aspects of my personality I don’t like” and “I’m disapproving and judgmental about my own flaws and inadequacies.”

##### Mindfulness

Five Facet Mindfulness Questionnaire [FFMQ-15; (80); Cronbach’s Alpha=0.81] is a fifteen-item scale that participants rate on a scale from 1 = *never or very rarely true* to 5 = *very often or always true* depending on how their level of agreement with the statements. Examples of items include, “I’m good at finding words to describe my feelings” and “I do jobs or tasks automatically without being aware of what I’m doing.”

##### Adaptive Coping

The Brief COPE scale has 28 items that assess tendencies for using adaptive and maladaptive coping strategies [(81); Cronbach’s Alpha=0.81] Items are rated on a scale from 1 = *I haven’t been doing this at all* to 4 = *I’ve been doing this a lot.* Examples of items include, “I’ve been giving up trying to deal with it” and “I’ve been trying to come up with a strategy about what I should do.”

##### Maladaptive Coping

See *Adaptive Coping*, above (Cronbach’s Alpha=0.75).

##### Optimism

The Life Orientation Test- Revised [LOT-R; (82); Cronbach’s Alpha=0.852] measures optimism. This test includes 10 items rated on a 5-point scale from 1 = *I disagree a lot* to 5 = *I agree a lot*. Examples of items include, “ If something can go wrong for me, it will” and “In uncertain times, I usually expect the best.”

##### Self-Esteem

The Single Item Self-Esteem Scale [SISE; (83)] is a single item measure of the construct of self-esteem that has been validated against the well-known Rosenberg Self-Esteem Scale. Scores range from 1 - 5 with low self-esteem indicated by a score of 1.

##### Social Connectedness

The Social Connectedness Scale Revised [SCS-R; (84); Cronbach’s Alpha=0.94] was used to measure social connectedness. Items were rated on a scale ranging from 1 = *strongly disagree* to 6 = *strongly agree*. Sample items include, “I am able to connect with other people”, “I see myself as a loner”, and “I feel like an outsider.”

#### Physical Health and Sleep

Participants answered two questions: “In general, would you say your health is…?” Items were rated on a scale 1 = *poor* to 5 = *excellent.* And “Over the last 2 weeks, how many days have you had trouble falling asleep, staying asleep, or sleeping too much?” Items were rated on a scale from 1 (day) to 14 (days). These assessments were also modeled on the CDC and RAND questionnaires mentioned under “Mental Health.”

#### Acceptability Assessments

At T2 (post-test), students answered 2 questions to which they could answer “yes,” “no,” or “maybe”: 1) Would you recommend that your university provide students with this workshop?, 2) Would you recommend this workshop to a friend?

### Analyses

**Figure 2:**
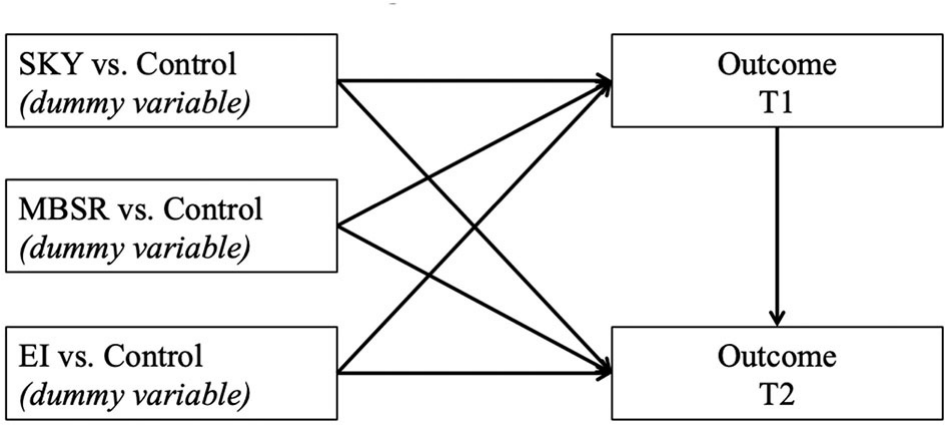
Regressions predicting outcomes at T2.

The correlations between variables can be found in **Table 1**. Cronbach’s alphas for each of the measures at each of time points can be found in Table A in **Supplementary Materials**. Treatment effects were tested with autoregressive models. Dummy variables were used to compare each of the three treatment groups to the control group, which was used as the reference group in the dummy variables (see **Figure 2**). The outcomes at T1 (pre-test) and T2 (post-test) were regressed on the dummy variables representing the effects of the three treatment groups, compared to the control group. The outcome at T2 was regressed on the outcome at T1 to control for T1 levels. All regressions were conducted in Mplus [Version 8.1; (85)], using a robust estimator (Maximum Likelihood Robust, MLR) due to non-normal distributions and outliers and Full Information Maximum Likelihood (FIML) estimation for missing data. This regression-based approach was selected over repeated measures MANOVAs to address the non-normality of the outcome variables, particularly outliers, and the problem of missing data from dropouts.

**Table 1:** Inter-correlations of outcome measures.

	1	2	3	4	5	6	7	8	9	10	11	12	13	14	15	16	17	18	19	20	21	22	23	24	25
1. Burnout	1	0.60*	0.45*	0.35*	0.27*	0.40*	0.33*	-0.36*	-0.08	-0.46*	-0.26*	-0.26*	-0.08	-0.41*	-0.32*	-0.28*	-0.29*	-0.36*	-0.34*	-0.22*	-0.39*	-0.32*	-0.45*	-0.31*	0.32*
2. Stress	0.57*	1	0.73*	0.49*	0.46*	0.66*	0.55*	-0.46*	-0.25*	-0.61*	-0.24*	-0.36*	-0.06	-0.53*	-0.49*	-0.45*	-0.35*	-0.61*	-0.53*	-0.30*	-0.56*	-0.46*	-0.51*	-0.32*	0.36*
3. Distress	0.43*	0.75*	1	0.44*	0.51*	0.74*	0.60*	-0.54*	-0.40*	-0.61*	-0.27*	-0.39*	-0.14	-0.65*	-0.47*	-0.53*	-0.42*	-0.61*	-0.57*	-0.19*	-0.57*	-0.54*	-0.56*	-0.33*	0.31*
4. Depression	0.37*	0.59*	0.45*	1	0.12	0.29*	0.26*	-0.49*	-0.29*	-0.36*	-0.22*	-0.51*	-0.09	-0.50*	-0.73*	-0.60*	-0.48*	-0.39*	-0.47*	-0.40*	-0.46*	-0.55*	-0.46*	-0.22*	0.25*
5. Anxiety	0.15	0.39*	0.38*	0.37*	1	0.62*	0.48*	-0.18*	-0.11	-0.29*	-0.07	-0.11	-0.03	-0.23*	-0.15*	-0.20*	-0.11	-0.32*	-0.30*	-0.03	-0.28*	-0.18*	-0.27*	-0.16*	0.39*
6. Negative affect	0.35*	0.68*	0.74*	0.22*	0.54*	1	0.60*	-0.44*	-0.24*	-0.48*	-0.14	-0.33*	-0.05	-0.53*	-0.26*	-0.40*	-0.22*	-0.51*	-0.48*	-0.08	-0.50*	-0.38*	-0.47*	-0.23*	0.38*
7. Maladaptive coping	0.22*	0.51*	0.64*	-0.49*	0.40*	0.53*	1	-0.36*	-0.33*	-0.47*	-0.21*	-0.23*	-0.04	-0.46*	-0.29*	-0.36*	-0.34*	-0.56*	-0.51*	-0.15*	-0.50*	-0.37*	-0.49*	-0.25*	0.34*
8. Life Satisfaction	-0.30*	-0.46*	-0.42*	-0.56*	-0.03	-0.26*	0.53*	1	0.29*	0.53*	0.17*	0.51*	0.16*	0.64*	0.42*	0.58*	0.50*	0.46*	0.49*	0.22*	0.60*	0.60*	0.61*	0.39*	-0.26*
9. RYFF Autonomy	-0.14	-0.30*	-0.32*	-0.34*	-0.06	-0.18*	-0.27*	0.42*	1	0.34*	0.22*	0.15*	0.10	0.43*	0.35*	0.28*	0.26*	0.42*	0.41*	0.23*	0.30*	0.48*	0.33*	0.20*	-0.09
10. RYFF Environment	-0.46*	0.62*	-0.46*	0.30*	-0.42*	-0.26*	0.45*	0.33*	0.33*	1	0.30*	0.34*	0.29*	0.56*	0.50*	0.45*	0.41*	0.52*	0.52*	0.31*	0.54*	0.47*	0.55*	0.44*	-0.34*
11. RYFF Growth	-0.14	-0.30*	-0.20*	-0.38*	-0.01	-0.21*	-0.22	0.38	0.30*	0.39*	1	0.34*	0.24*	0.35*	0.33*	0.31*	0.37*	0.23*	0.28*	0.21*	0.34*	0.26*	0.19*	0.29*	-0.13
12. RYFF Relations	-0.20*	-0.33*	-0.29*	-0.43*	-0.08	-0.30*	-0.19	0.44*	0.33*	0.35*	0.39*	1	0.13	0.60*	0.44*	0.75*	0.57*	0.46*	0.44*	0.33*	0.48*	0.41*	0.44*	0.22*	-0.29*
13. RYFF Purpose	0.05	-0.12	-0.09	-0.09	-0.20*	-0.07	-0.18*	0.32*	0.17	0.25*	0.26*	0.28*	1	0.22*	0.26*	0.19*	0.21*	0.02	0.18*	0.20*	0.11	0.15*	0.15*	0.18*	0.03
14. RYFF Self-acceptance	-0.23*	-0.47*	-0.48*	-0.59*	-0.15	-0.39*	-0.27*	0.64*	0.47*	0.46*	0.47*	0.53*	0.28*	1	0.50*	0.64*	0.56*	0.61*	0.60*	0.37*	0.59*	0.65*	0.58*	0.38*	-0.34*
15. Positive affect	-0.35*	-0.47*	-0.37*	-0.58*	-0.10	-0.20*	-0.39*	0.42*	0.27*	0.46*	0.38*	0.28*	0.19*	0.47*	1	0.50*	0.47*	0.35*	0.48*	0.42*	0.45*	0.50*	0.50*	0.43*	-0.30*
16. Connectedness	-0.22*	-0.42*	-0.44*	-0.19*	-0.15	-0.36*	-0.20*	0.47*	0.28*	0.37*	0.47*	0.76*	0.29*	0.61*	0.46*	1	0.59*	0.56*	0.56*	0.43*	0.55*	0.49*	0.53*	0.31*	-0.23*
17. Gratitude	-0.20*	-0.36*	-0.31*	-0.46*	-0.11	-0.29*	-0.01	0.53*	0.25*	0.38*	0.50*	0.55*	0.25*	0.51*	0.37*	0.60*	1	0.45*	0.51*	0.39*	0.49*	0.45*	0.46*	0.27*	-0.17*
18. Self-Compassion	-0.15	-0.49*	-0.56*	-0.50*	-0.17	-0.51*	-0.29*	0.33*	0.31*	0.39*	0.44*	0.35*	0.03	0.50*	0.41*	0.47*	0.46*	1	0.65*	0.40*	0.58*	0.56*	0.56*	0.27*	-0.34*
19. Mindfulness	-0.28*	-0.50*	-0.49*	-0.44*	-0.28*	-0.42*	-0.51*	0.40*	0.33*	0.43*.	0.51*	0.41*	0.24*	0.54*	0.52*	0.53*	0.50*	0.66*	1	0.42*	0.57*	0.55*	0.63*	0.36*	-0.36*
20. Adaptive Coping	-0.07	-0.24*	-0.08	-0.51*	0.08	0.02	-0.42*	0.37*	0.26*	27*	0.47*	0.37*	0.29*	0.47*	0.47*	0.44*	0.45*	0.47*	0.55*	1	0.35*	0.29*	0.23*	0.26*	-0.16*
21. Optimism	-.16	-0.47*	-0.50*	-0.39*	-0.25*	-0.43*	-0.43*	0.45*	0.27*	0.36*	0.45*	0.37*	0.24*	0.50*	0.27*	0.46*	0.49*	0.58*	0.56*	0.33*	1	0.59*	0.55*	0.40*	-0.31*
22. Self-esteem	-0.26*	-0.51*	-0.45*	-0.58*	-0.15	-0.28*	-0.27*	0.59*	0.38*	0.49*	0.36*	0.29*	0.18*	0.65*	0.57*	0.39*	0.34*	0.46*	0.52*	0.41*	0.46*	1	0.61*	0.41*	-0.28*
23. Mental Health	-0.42*	-0.57*	-0.50*	-0.28*	-0.33*	-0.47*	0.02	0.54*	0.21*	0.56*	0.23*	0.35*	0.29*	0.48*	0.49*	0.44*	0.33*	0.39*	0.44*	0.27*	0.39*	0.54*	1	0.44*	-0.39*
24. Physical Health	-0.24*	-0.21*	-0.17	0.15	-0.12	-0.07	-0.47*	0.25*	0.36*	0.40*	0.21*	0.22*	0.19*	0.42*	0.40*	0.25*	0.15	0.24*	0.27*	0.23*	0.17*	0.44*	0.31*	1	-0.25*
25. Sleep Problems	0.32*	0.31*	0.30*	0.37*	0.27*	0.24*	-0.07	-0.19*	-0.04	-0.23*	-0.17*	-0.02	-0.10	-0.12	-0.16	-0.16	-0.24*	-0.17	-0.26*	-0.11	-0.23*	-0.17	-0.34*	-0.12	1

*p ≤ 0.05.

Correlations above the diagonal indicate correlations between variables at T1 while correlation coefficients below the diagonal indicate correlations between variables at T2.

Since we had directed hypotheses for the comparisons of each intervention group to the control group, we report one-tailed p-values. Due to the multiple comparisons, the p-values were Bonferroni-corrected for 72 hypotheses; 24 outcomes multiplied with 3 intervention dummy variables. The adjusted p-value needed to be <= 0.000641026 (or 0.001, since Mplus only reports three decimal points).

## Results

### Treatment Effects at T2

To estimate treatment effects, we conducted autoregressive regression models. In these models, treatment groups were represented by dummy variables, comparing each treatment group to the reference control group. The outcomes at T1 and T2 were regressed on the three treatment group dummy variables, and the outcome at T1 was regressed on the T1-level of itself, to control for baseline levels. Due to the relatively small sample size, we conducted a separate regression for each outcome. **Figure 1** shows examples of the regression models conducted (for treatment effects at T2). The detailed results for all regressions that examined treatment effects, including p-values, are reported in **Tables 2**–**4**. All effects below are in comparison to the control group and controlling for T1:

**Table 2:** From T1 to T2: Regressions for mental health outcomes regressed on groups.

Predictors	Outcome Time 1				Outcome Time 2			
	*B*	*SE(B)*	β	*p*	*B*	*SE(B)*	β	*p*
**Burnout T1**	–	–	–	–	0.18	0.09	0.18	0.027
Sky vs. Control^a^	0.32	0.14	0.18	0.014	−0.41	0.17	−0.23	0.006
MBSR vs. Control^b^	0.33	0.14	0.20	0.007	−0.32	0.17	−0.20	0.023
EI vs. Control^c^	0.52	0.14	0.27	0.001	−0.28	0.21	−0.15	0.089
*R^2^*	.06	–	–	0.042	.06	–	–	0.080
**Stress T1**	–	–	–	–	0.36*	0.09	0.35	0.000
Sky vs. Control^a^	0.30	0.13	0.21	0.009	−0.47*	0.11	−0.32	0.000+
MBSR vs. Control^b^	0.30	0.11	0.21	0.005	−0.21	0.14	−0.15	0.066
EI vs. Control^c^	0.37	0.13	0.24	0.002	−0.2	0.17	−0.12	0.131
*R^2^*	.06	–	–	0.043	.17	–	–	0.003
**Distress T1**	–	–	–	–	0.34*	0.07	0.41	0.000
Sky vs. Control^a^	0.17	0.17	0.09	0.148	−0.24	0.12	−0.16	0.016
MBSR vs. Control^b^	0.09	0.14	0.05	0.269	−0.16	0.13	0.11	0.122
EI vs. Control^c^	0.20	0.18	0.10	0.128	0.08	0.18	−0.05	0.333
*R^2^*	0.01	–	–	0.253	.18*	–	–	0.002
**Depression T1**	–	–	–	–	0.47*	0.08	0.48	0.000
Sky vs. Control^a^	0.29	0.15	0.16	0.028	−0.50*	0.14	−0.29	0.000+
MBSR vs. Control^b^	0.43	0.25	0.25	0.002	−0.13	0.14	−0.08	0.170
EI vs. Control^c^	0.30	0.26	0.15	0.029	−0.19	0.17	−0.10	0.139
*R^2^*	.05	–	–	0.069	.27*	–	–	0.000
**Anxiety T1**	–	–	–	–	0.51*	0.06	0.63	0.000
Sky vs. Control^a^	0.20	0.13	0.14	0.055	−0.14	0.09	−0.12	0.050
MBSR vs. Control^b^	0.14	0.11	0.10	0.099	−0.12	0.09	−0.11	0.092
EI vs. Control^c^	0.11	0.13	0.07	0.202	−0.16	0.1	−0.13	0.045
*R^2^*	.02	–	–	0.191	.39*	–	–	0.000
**Mental Health T1**	–	-	–	–	0.63*	0.06	0.64	0.000
Sky vs. Control^a^	−0.41	0.23	−0.15	0.034	0.66*	0.17	0.25	0.000+
MBSR vs. Control^b^	−0.59	0.21	−0.24	0.002	−0.12	0.21	−0.05	0.285
EI vs. Control^c^	−0.72	0.25	−0.25	0.002	0.47	0.19	0.16	0.007
*R^2^*	0.06	–	–	0.044	0.47*	–	–	0.000

+ : *p*(one-tailed) ≤ 0.000 (Bonferroni-adjusted); * : *p*(one-tailed) ≤ 0.05; ^a^coded 1, SKY treatment group; 0, all other groups; ^b^coded 1, MBSR treatment group; 0, all other groups; ^c^coded 1, EI treatment group, 0, all other groups.

**Table 3:** From T1 to T2: Regressions for well-being outcomes regressed on groups.

Predictors	Outcome Time 1				Outcome Time 2			
	*B*	*SE(B)*	β	*p (1-tailed)*	*B*	*SE(B)*	β	*p (1-tailed)*
**Life Satisfaction T1**	–	-	–	–	0.57*	0.06	0.71	0.000
Sky vs. Control^a^	−0.70	0.24	−0.24	0.001	0.35	0.17	0.15	0.017
MBSR vs. Control^b^	−0.06	0.21	−0.22	0.002	0.12	0.16	0.05	0.237
EI vs. Control^c^	−0.61	0.27	−0.20	0.011	0.38	0.18	0.15	0.012
*R^2^*	0.06	–	–	0.032	0.48*	–	–	0.000
**Ryff Autonomy T1**					0.59*	0.05	0.69	0.000
Sky vs. Control^a^	0.05	0.26	0.02	0.418	0.38	0.18	0.14	0.016
MBSR vs. Control^b^	−0.05	0.24	−0.02	0.427	0.38	0.17	0.15	0.014
EI vs. Control^c^	0.03	0.28	0.01	0.457	0.05	0.25	0.02	0.416
*R^2^*	.00			0.426	.50*			0.000
**Ryff Environment T1**					0.49*	0.08	0.51	0.000
Sky vs. Control^a^	−0.51	0.23	−0.19	0.014	0.68	0.21	0.27	0.001
MBSR vs. Control^b^	−0.60	0.20	−0.24	0.001	0.20	0.21	0.08	0.167
EI vs. Control^c^	−0.77	0.24	−0.27	0.001	0.40	0.23	0.15	0.040
*R^2^*	.07			0.024	.28*			0.000
**Ryff Growth T1**					0.43*	0.07	0.50	0.000
Sky vs. Control^a^	−0.02	0.14	−0.01	0.448	0.22	0.13	0.16	0.043
MBSR vs. Control^b^	−0.01	0.13	0.00	0.481	0.11	0.12	0.08	0.187
EI vs. Control^c^	−0.08	0.16	−0.05	0.304	0.28	0.11	0.18	0.005
*R^2^*	.00			0.393	.27*			0.001
**Ryff Relations T1**					0.64*	0.05	0.74	0.000
Sky vs. Control^a^	−0.11	0.27	−0.04	0.340	0.42	0.18	0.15	0.009
MBSR vs. Control^b^	−0.45	0.24	−0.15	0.030	0.09	0.18	0.03	0.318
EI vs. Control^c^	−0.41	0.29	−0.12	0.078	0.15	0.17	0.05	0.198
*R^2^*	.02			0.147	.57*			0.000
**Ryff Purpose T1**					0.57*	0.08	0.57	0.000
Sky vs. Control^a^	−0.05	0.19	−0.02	0.406	0.05	0.18	0.02	0.384
MBSR vs. Control^b^	−0.08	0.17	−0.04	0.320	−0.12	0.19	−0.06	0.253
EI vs. Control^c^	−0.37	0.22	−0.15	0.044	0.12	0.21	0.05	0.276
*R^2^*	.02			0.195	.33*			0.000
**Ryff Self-Acceptance**					0.44*	0.05	0.56	0.000
Sky vs. Control^a^	−0.36	0.23	−0.13	0.056	0.57*	0.17	0.25	0.001
MBSR vs. Control^b^	−0.44	0.21	−0.17	0.018	0.28	0.20	0.13	0.079
EI vs. Control^c^	−0.40	0.28	−0.13	0.081	0.45	0.18	0.18	0.008
*R^2^*	.02			0.118	.33*			0.000
**Positive affect T1**	–	–	–	–	0.56*	0.07	0.54	0.000
Sky vs. Control^a^	−0.10	0.14	−0.06	0.242	0.51*	0.15	0.30	0.000+
MBSR vs. Control^b^	−0.31	0.13	−0.20	0.010	0.41	0.13	0.26	0.001
EI vs. Control^c^	−0.20	0.15	−0.11	0.086	0.16	0.13	0.09	0.104
*R^2^*	.03	–	–	0.111	.35*	–	–	0.000
**Connectedness T1**	–	–	–	–	0.65*	0.06	0.75	0.000
Sky vs. Control^a^	−0.22	0.2	−0.10	0.131	0.39*	0.11	0.21	0.000+
MBSR vs. Control^b^	−0.26	0.17	−0.13	0.063	0.04	0.13	0.03	0.369
EI vs. Control^c^	−0.43	0.19	−0.18	0.011	0.26	0.13	0.13	0.020
*R^2^*	.03	–	–	0.212	.57*	–	–	0.000
**Gratitude T1**	–	–	–	–	0.57*	0.06	0.69	0.000
Sky vs. Control^a^	−0.60	0.20	−0.03	0.384	0.27	0.13	0.14	0.016
MBSR vs. Control^b^	−0.30	0.17	−0.14	0.041	−0.02	0.14	−0.10	0.445
EI vs. Control^c^	−0.22	0.22	−0.09	0.159	0.27	0.15	0.13	0.027
*R^2^*	.02	–	–	0.179	.51*	–	–	0.000
**Self-Compassion T1**	–	–	–	–	0.47*	0.08	0.52	0.000
Sky vs. Control^a^	−0.26	0.17	−0.14	0.063	0.35	0.14	0.20	0.006
MBSR vs. Control^b^	−0.29	0.14	−0.16	0.020	0.12	0.13	0.07	0.197
EI vs. Control^c^	−0.20	0.18	−0.10	0.125	0.25	0.16	0.14	0.056
*R^2^*	.02	–	–	0.139	.29*	–	–	0.001
**Mindfulness T1**	–	–	–	–	0.58*	0.07	0.62	0.000
Sky vs. Control^a^	−0.09	0.11	−0.07	0.201	0.31*	0.09	0.26	0.000+
MBSR vs. Control^b^	−0.18	0.1	−0.15	0.038	0.19	0.10	0.18	0.018
EI vs. Control^c^	−0.33	0.1	−0.25	0.001	0.36*	0.36	0.28	0.000+
*R^2^*	.05	–	–	0.047	.40*	–	–	0.000
**Adaptive Coping T1**	–	–	–	–	0.55*	0.07	0.53	0.000
Sky vs. Control^a^	−0.02	0.12	−0.02	0.423	0.26	0.12	0.20	0.012
MBSR vs. Control^b^	−0.01	0.1	−0.01	0.462	0.21	0.11	0.17	0.025
EI vs. Control^c^	−0.00	0.12	0.01	0.045	0.25	0.12	0.17	0.013
*R^2^*	.00			0.242	.32			0.000
**Optimism T1**					0.50*	0.09	0.56	0.000
Sky vs. Control^a^	−0.30	0.16	−0.16	0.058	0.21	0.14	0.12	0.135
MBSR vs. Control^b^	−0.32	0.14	−0.19	0.020	0.17	0.13	0.11	0.186
EI vs. Control^c^	−0.29	0.16	−0.15	0.078	0.13	0.14	0.08	0.345
*R^2^*	.03			0.176	.31*			0.001
**Self-esteem T1**					0.58*	0.05	0.74	0.000
Sky vs. Control^a^	−0.44	0.23	−0.15	0.053	0.41	0.14	0.18	0.003
MBSR vs. Control^b^	−0.48	0.23	−0.18	0.038	0.07	0.17	0.03	0.670
EI vs. Control^c^	−0.31	0.27	−0.10	0.250	0.21	0.14	0.08	0.141
*R^2^*	.03			0.235	.55*			0.000
**Negative affect T1**	–	–	–	–	0.38*	0.09	0.42	0.000
Sky vs. Control^a^	0.26	0.14	0.16	0.032	−0.29	0.12	−0.19	0.006
MBSR vs. Control^b^	0.22	0.13	0.14	0.040	−0.13	0.14	−0.10	0.176
EI vs. Control^c^	0.28	0.16	0.16	0.033	−0.26	0.15	−0.17	0.035
*R^2^*	.03	–	–	0.123	.19*	–	–	0.004
**Maladaptive Coping T1**	–	–	–	–	0.47	−0.10	0.50	0.000
Sky vs. Control^a^	0.14	0.09	0.14	0.047	−0.18*	0.08	−0.18	0.006
MBSR vs. Control^b^	0.15	0.08	0.15	0.028	−0.11	0.79	−0.12	0.076
EI vs. Control^c^	0.20	0.10	0.18	0.018	−0.20*	0.11	−0.19	0.042
*R^2^*	0.03	–	–	0.104	.26*	–	–	0.001

+ : *p*(one-tailed) ≤ 0.000 (Bonferroni-adjusted); * : *p*(one-tailed) ≤ 0.05; ^a^coded 1, SKY treatment group; 0, all other groups; ^b^coded 1, MBSR treatment group; 0, all other groups; ^c^coded 1, EI treatment group; 0, all other groups.

**Table 4:** From T1 to T2: Regressions for health well-being outcomes regressed on groups.

	Time 1				Time 2			
	*B*	*SE(B)*	*β*	*p*	*B*	*SE(B)*	*β*	*p*
**Physical Health T1**	–	–	–	–	0.68	0.05	0.77	0.000
Sky vs. Control^a^	−0.45	0.20	−0.18	0.011	0.15	0.14	0.07	0.144
MBSR vs. Control^b^	−0.45	0.20	−0.20	0.010	0.07	0.13	0.03	0.310
EI vs. Control^c^	−0.74	0.22	−0.28	0.000	0.04	0.15	0.02	0.393
*R^2^*	0.07	–	–	0.029	.58*	–	–	0.000
**Sleep Problems T1**	–	–	–	–	0.27*	0.08	0.33	0.000
Sky vs. Control^a^	1.42	0.79	0.15	0.036	−1.21	0.64	−0.16	0.026
MBSR vs. Control^b^	1.78	0.78	0.20	0.011	0.21	0.80	0.03	0.394
EI vs. Control^c^	0.59	0.78	0.06	0.225	−0.72	0.78	−0.09	0.178
*R^2^*	0.03	–	–	0.102	0.14	–	–	0.012

+ : *p*(one-tailed) ≤ 0.000 (Bonferroni-adjusted); * : *p*(one-tailed) ≤ 0.05; ^a^ coded 1 = SKY treatment group, 0 = all other groups; ^b^ coded 1 = MBSR treatment group, 0 = all other groups; ^c^coded 1 = EI treatment group, 0 = all other groups.

#### Effects of SKY

The SKY group scored significantly higher than the control group on three mental health outcomes at T2, while controlling for the T1 levels in these three outcomes: depression (MASQ; β = −0.29), mental health (β = 0.25), and stress (PSS-10; β = −0.32). Moreover, the SKY group scored significantly higher than the control group on three outcomes of psychological thriving: mindfulness (FFMQ-15; β = 0.26), positive affect (PANAS; β = 0.30), and social connectedness (SCS-R; β = 0.21).

#### Effects of EI

The EI group scored significantly higher than the control group on one aspect of psychological thriving: mindfulness (FFMQ-15; β = 0.28).

#### Effects of MBSR

The MBSR group did not differ in any outcome from the control group at T2. (However, see below for findings warranting replications with larger samples).

### Uncorrected Treatment Effects at T2

The strict Bonferroni correction, used to correct for the multiple comparisons, led us to decrease the acceptable p-value from the conventional 0.050 to 0.000. However, given our small sample size, the likelihood of detecting effects with such a strict threshold for significance is small. There were several findings of effects that were significant at the *p* ≤ 0.05 level that became insignificant after the alpha adjustment. We suggest that these effects be examined in future studies with larger samples as these effects do warrant further investigation. These were the differences between intervention groups and the control group at T2 that were significant at the *p* ≤ 0.05-level but not at the adjusted *p* ≤ 0.000-level were:

#### Effects of SKY

Twelve outcomes warranting replication with larger samples: environment-related well-being (Ryff; β = 0.27); self-acceptance (Ryff; β =0.25); burnout (β = −0.23), self-compassion (SCS-SF; β = 0.20), adaptive coping (COPE; β = 0.20), negative affect (PANAS; β = −0.19), self-esteem (β = 0.18), maladaptive coping (COPE; β = −0.18), distress (MASQ; β = −0.16), sleep problems (β = −0.16), life satisfaction (SWLS; β = 0.15), relationship-related well-being (Ryff; β = 0.15); autonomy (Ryff; β = 0.14), gratitude (GQ-6; β = 0.14), anxiety (MASQ; β = −0.12).

#### Effects of EI

Nine outcomes warranting replication with larger samples: maladaptive coping (COPE; β = −0.19), self-acceptance (RYFF; β =0.18), growth-related well-being (RYFF; β =0.18), adaptive coping (COPE; β = 0.17), negative affect (PANAS; β = −0.17), mental health (β = 0.16), life satisfaction (SWLS; β = 0.15), social connectedness (SCS-R; β = 0.13), anxiety (MASQ; β = −0.13), and gratitude (GQ-6; β = 0.13).

#### Effects of MBSR

Five outcomes warranting replication with larger samples: positive affect (PANAS; β = 0.26), burnout (β = −0.20), autonomy (Ryff; β =0.15), mindfulness (FFMQ-15; β = 0.18), and adaptive coping (COPE; β = 0.17).

We also compared the intervention groups with each other on all the outcomes for which multiple interventions showed significant differences (*p* ≤ 0.05) to the control group (see Tables F-N in **Supplementary Materials**). There was no significant difference between the intervention groups on any of these outcomes after multiple comparison correction using the strict Bonferroni correction.

### Robustness Check

We used nonparametric Mann-Whitney-U-tests for pairwise comparisons between each treatment group and the control group in the change score T2 minus T1 on the outcome variables. The Mann-Whitney-U test indicated largely the same results as the aforementioned regressions. Please see the Tables in the **Supplementary Materials** detailing results of the Mann-Whitney-U tests for the comparisons of the change scores from T1 to T2 (Table C). Since the regression analyses did not directly assess *change* from T1 to T2, we additionally examined whether the change was significant by comparing T1 and T2 for each outcome variable with paired-samples t-test (Table D, **Supplementary Materials**). Attrition analyses can also be found in the **Supplementary Material** (Table E).

### Acceptance of the Intervention

Similarly to the pilot study, the majority of students in all three groups said they would recommend the interventions to their university and friends, suggesting good acceptance of the programs among the students. See **Figure 1**, **Supplementary Materials**.

## Discussion

In sum, compared to the control group and controlling for baseline levels in the outcomes and for multiple comparisons, the SKY group benefited six outcomes (depression, stress, mental health, positive affect, mindfulness, and social connectedness), the EI group benefited one outcome (mindfulness), and the MBSR benefited none.

SKY’s wide range of results may be explained by its multi-component and comprehensive curriculum including a number of different factors that prior research suggests can improve mental health and psychological thriving: positive psychology (86), yoga/breathing/meditation (87–89), and community service (90). Modulation of respiration, in particular, has been linked to improvements in neuro-cognitive function (91, 92). Since cognitive deficits are often observed in mental health conditions (93), breathing exercises may impact mental health and psychological thriving via improved cognitive function, such as enhanced attention (94). Breathing techniques have also been found to enhance autonomic, cerebral and psychological flexibility related to emotional control and psychological well-being in healthy participants (95).

The EI program impacted mindfulness, perhaps because of the program’s focus on self-awareness and emotional awareness in particular (54).

MBSR’s lack of significant findings were surprising given a substantial body of research suggesting its potential benefits for a wide range of populations [e.g., (62, 80)]. Meta-analyses on MBSR, however, are mixed and suggest a range of effect sizes from small to large depending on the measured construct [e.g., (96)]. MBSR’s comparatively modest impact in the current study’s population may be explained by the fact that MBSR is designed for a general community audience whereas SKY and EI programs are specifically tailored for university students. Further studies are required to determine the impact of potential adaptations of the MBSR curriculum for university students.

While the MBSR intervention showed no significant difference from the control group at T2 in any outcome with the Bonferroni-adjusted p-value of 0.000, there were several outcomes for which the difference between the MBSR and the control group would have been significant at the conventional level of p ≤ .05. The same is true for the SKY group and the MBSR group. Because the current study is underpowered to detect effects at the stricter level of significance, this study warrants replication with larger sample sizes. Based on these indications, it is worth investigating whether all of the interventions might show additional benefits.

The American Council for Education emphasizes the cost-benefit analyses of offering mental health programming to reduce costs related to dropout and earnings (29). Counseling expenses for an average public 4-year college or university serving 15,000-20,000 students, with an average staff of 10 full-time staff, are around $1,560,000 (including salary, benefits, operational costs and professional development) (5). Programs like SKY, EI or MBSR can be taught in a large group format by trainers hired as lecturers. The cost of hiring lecturers per year is low [average salary during the academic year 2016–17 was $58,749; (97)]. Given that these programs offer empirically validated education that leads to observable improvements in well-being and mental health, and given the affordability of their delivery, universities could stand to benefit financially from including them in their curricula.

## Limitations and Future Directions

Although effect sizes were consistent and in expected directions across many outcome variables, group sizes were small. Due to randomization and dropout, group sizes were also uneven. There was also low internal consistency of the dimensions that make up the Ryff Scale of Psychological Well-Being.

Replications with larger samples and group sizes are recommended. Moreover, future research should include clinical and biological measures in addition to self-report.

Whereas the 10-hour pilot study showed no effects, the 30-hour program with required at-home practice did. The previous null findings may indicate that the amount of training hours used in this study should not be reduced in future studies and in trainings implemented in colleges and universities. Further research could also examine the replicability of these findings for online delivery of these programs.

With the modest effects of psychoeducation-only programs for students (as discussed in the introduction), the integrative format of the workshops studied here, which include both skill-building and group dynamics, may be critical for the effectiveness of the programs (34). In addition, future research should investigate mechanisms responsible for the effects of SKY and EI and interactive effects of different curricula components.

It is unclear whether the results would differ if the program structure and incentivization process were altered. Future research may also consider the role of personality traits or a natural propensity toward well-being on outcomes.

Participants were restricted to undergraduate students from a particularly competitive university in the United States. Replicating the study across a variety of campuses and cultural contexts and including additional student populations, such as graduate students, would provide a more comprehensive assessment of the interventions. Moreover, the findings should be considered in relation to the large number of outcome measures, which necessitated correction for multiple comparisons.

Inactive control groups make it difficult to determine whether the effect of a given intervention is due to content alone (35). Future studies should include an active control group.

In sum, this study is one of the first to offer an evaluation of several recommendable and effective protocols for university administrators. Given the growing mental health crisis among college-aged students and increasing demand for university campus mental health services, there is an urgent need for cost-effective and preventative ways to address the problem. Providing preventative skills-building psychoeducational programs such as SKY and EI, as a supplement to standard care, may help buffer students’ mental health and well-being while providing relief from the financial and counseling burden on universities.

## Data Availability Statement

The datasets generated for this study are available on request to the corresponding author.

## Ethics Statement

The studies involving human participants were reviewed and approved by Yale Human Research Protection Program. The patients/participants provided their written informed consent to participate in this study.

## Author Contributions

ES, CB, and MB designed the pilot study and main study, participated in recruitment and data collection, consulted on data analysis, and wrote up the manuscript. JM conducted most of the data analyses and contributed to the manuscript. LH participated in recruitment, data collection and manuscript write-up. DN collected the pilot study data, consulted on the main study, and contributed to the manuscript.

## Funding

This research was funded by an anonymous donor to the Yale Center for Emotional Intelligence. This project was in part also supported by a Jacobs Foundation Early Career Research Fellowship to JM.

## Conflict of Interest

The authors declare that the research was conducted in the absence of any commercial or financial relationships that could be construed as a potential conflict of interest.
