# Supplementary material for: Promoting Mental Health and Psychological Thriving in University Students: A Randomized Controlled Trial of Three Well-Being Interventions
Source: Front Psychiatry. 2020 Jul 15;11:590. doi: 10.3389/fpsyt.2020.00590 (PMC7373803; doi:10.3389/fpsyt.2020.00590)
Supplement: Supplementary file 1 [file DataSheet_1.docx]

Supplementary Material

**Table A:** Overall descriptives for all measures at T1 (pretest; N = 189) & T2 (posttest1; N = 131)

|  | Time 1 | | | Time 2 | | |
| --- | --- | --- | --- | --- | --- | --- |
| **Variable** | *Mean* | *SD* | *α* | *Mean* | *SD* | *α* |
| **Mental Health** |  |  |  |  |  |  |
| Burnout | 2.36 | 0.76 | - | 2.37 | 0.75 | - |
| Stress | 2.86 | 0.61 | 0.85 | 2.59 | 0.62 | 0.87 |
| MASQ Distress | 2.62 | 0.79 | 0.88 | 2.20 | 0.65 | 0.85 |
| MASQ Depression | 2.93 | 0.77 | 0.91 | 2.74 | 0.74 | 0.91 |
| MASQ Anxiety | 1.80 | 0.61 | 0.8 | 1.61 | 0.5 | 0.77 |
| Mental Health | 2.98 | 1.14 | - | 3.37 | 1.12 | - |
| **Well-Being** |  |  |  |  |  |  |
| Life satisfaction | 4.83 | 1.22 | 0.82 | 5.44 | 0.98 | 0.82 |
| Ryff Autonomy | 4.81 | 1.32 | 0.69 | 5.04 | 1.12 | 0.68 |
| Ryff Environment | 4.44 | 1.14 | 0.55 | 4.88 | 1.06 | 0.61 |
| Ryff Growth | 6.33 | 0.71 | 0.41 | 6.40 | 0.61 | 0.48 |
| Ryff Relations | 5.20 | 1.36 | 0.66 | 5.51 | 1.17 | 0.59 |
| Ryff Purpose | 5.60 | 0.97 | 0.35 | 5.46 | 0.99 | 0.33 |
| Ryff Self-acceptance | 5.41 | 1.22 | 0.72 | 5.71 | 0.95 | 0.56 |
| Pleasant Emotions | 3.00 | 0.70 | 0.89 | 3.09 | 0.72 | 0.9 |
| Social Connectedness | 4.17 | 0.92 | 0.94 | 4.42 | 0.79 | 0.93 |
| Adaptive Coping | 2.72 | 0.55 | 0.81 | 2.81 | 0.57 | 0.84 |
| Gratitude | 5.93 | 0.99 | 0.86 | 6.15 | 0.82 | 0.79 |
| Self-Compassion | 2.77 | 0.80 | 0.89 | 3.20 | 0.72 | 0.87 |
| Mindfulness | 3.17 | 0.53 | 0.81 | 3.43 | 0.5 | 0.83 |
| Optimism | 3.34 | 0.78 | 0.85 | 3.56 | 0.69 | 0.81 |
| Self-esteem | 3.25 | 1.22 | - | 3.76 | 0.95 | - |
| Unpleasant Emotions | 2.24 | 0.70 | 0.87 | 2.00 | 0.62 | 0.87 |
| Maladaptive Coping | 1.92 | 0.45 | 0.75 | 1.77 | 0.4 | 0.73 |
| **Health Outcomes** |  |  |  |  |  |  |
| Physical Health | 3.25 | 1.03 | - | 3.47 | 0.91 | - |
| Sleep Problems | 5.09 | 4.01 | - | 3.55 | 3.25 | - |
|  |  |  |  |  |  |  |

**Table B**: Means and standard deviations by intervention group and measurement time point.

|  | **SKY** | | **MBSR** | | **EI** | | **Control** | |  |
| --- | --- | --- | --- | --- | --- | --- | --- | --- | --- |
| **Outcome Variable** | **Time 1** | **Time 2** | **Time 1** | **Time 2** | **Time 1** | **Time 2** | **Time 1** | **Time 2** | |
|  | **(N = 44)** | **(N = 29)** | **(N = 55)** | **(N = 34)** | **(N = 36)** | **(N = 21)** | **(N = 54)** | **(N = 47)** | |
| **Mental Health** |  |  |  |  |  |  |  |  | |
| Burnout | 2.41 (.73) | 2.21 (.68) | 2.42 (.71) | 2.26 (.71) | 2.61 (.84) | 2.33 (.80) | 2.09 (.71) | 2.55 (.78) | |
| Stress | 2.93(.68) | 2.34 (.47) | 2.92 (.56) | 2.59 (.67) | 3.01 (.61) | 2.63 (.68) | 2.64 (.58) | 2.72 (.58) | |
| MASQ Distress | 2.69 (.86) | 2.08 (.50) | 2.60 (.68) | 2.15 (.59) | 2.72 (.88) | 2.24 (.77) | 2.51 (.78) | 2.29 (.71) | |
| MASQ Depression | 2.97 (.72) | 2.43 (.70) | 3.11 (.78) | 2.88 (.79) | 2.98 (.72) | 2.81 (.70) | 2.68 (.78) | 2.80 (.71) | |
| MASQ Anxiety | 1.89 (.69) | 1.59 (.46) | 1.83 (.57) | 1.59 (.44) | 1.81 (.64) | 1.52 (.48) | 1.70 (.53) | 1.67 (.56) | |
| Mental Health | 3.00 (1.16) | 3.79 (.94) | 2.80 (1.01 | 2.94 (1.10) | 2.67 (1.22) | 3.33 (1.06) | 3.39 (1.16) | 3.45 (1.18) | |
| **Psychological Thriving** |  |  |  |  |  |  |  |  | |
| Satisfaction with Life | 4.58 (1.24) | 5.48 (.86) | 4.68 (1.6) | 5.29 (1.15) | 4.67 (1.39) | 5.46 (.65) | 5.28 (1.08) | 5.50 (1.06) | |
| Ryff Autonomy | 4.86 (1.37) | 5.31 (1.03) | 4.76 (1.40) | 5.21 (.96) | 4.83 (1.43) | 4.84 (1.31) | 4.08 (1.16) | 4.84 (1.18) | |
| Ryff Environment | 4.37 (1.23) | 5.21 (1.16) | 4.28 (1.05) | 4.79 (.94) | 4.11 (1.18) | 4.83 (1.27) | 4.88 (1.01) | 4.77 (.96) | |
| Ryff Growth | 6.33 (.70) | 6.51 (.67) | 6.33 (.68) | 6.35 (.59) | 6.27 (.80) | 6.57 (.51) | 6.35 (.70) | 6.31 (.63) | |
| Ryff Relations | 5.33 (1.48) | 5.77 (1.04) | 4.99 (1.32) | 5.27 (1.33) | 5.03 (1.14) | 5.35 (1.16) | 5.44 (1.19) | 5.58 (1.13) | |
| Ryff Purpose | 5.66 (1.01) | 5.54 (.92) | 5.62 (.96) | 5.28 (1.13) | 5.33 (1.50) | 5.33 (1.10) | 5.70 (.81) | 5.59 (.87) | |
| Ryff Self-acceptance | 5.34 (1.19) | 5.97 (.85) | 5.26 (1.16) | 5.60 (.93) | 5.31 (1.49) | 5.83 (.79) | 5.70 (1.06) | 5.57 (1.06) | |
| Pleasant Emotions | 3.05 (.72) | 3.37 (.75) | 2.85 (.67) | 3.11 (.73) | 2.95 (.69) | 2.95 (.69) | 3.16 (.72) | 2.96 (.73) | |
| Social Connectedness | 4.13 (1.03) | 4.71 (.62) | 4.10 (.83) | 4.23 (.82) | 3.95 (.82) | 4.28 (.63) | 4.36 (.95) | 4.44 (.90) | |
| Gratitude | 6.01 (1.09) | 6.34 (.74) | 5.78 (.93) | 5.94 (.89) | 5.86 (1.09) | 6.25 (.65) | 6.07 (.88) | 6.15 (.86) | |
| Self-Compassion | 2.69 (.88) | 3.37 (.82) | 2.66 (.73) | 3.15 (.74) | 2.75 (.87) | 3.26 (.51) | 2.94 (.76) | 3.12 (.71) | |
| Mindfulness | 3.22 (.57) | 3.59 (.45) | 3.13 (.52) | 3.40 (.56) | 2.98 (.46) | 3.50 (.32) | 3.31 (.54) | 3.31 (.54) | |
| Adaptive Coping | 2.71 (.61) | 2.90 (.63) | 2.72 (.51) | 2.86 (.61) | 2.74 (.56) | 2.92 (.40) | 2.73 (.55) | 2.68 (.55) | |
| Optimism | 3.26 (.86) | 3.65 (.72) | 3.24 (.76) | 3.54 (.71) | 3.27 (.81) | 3.53 (.61) | 3.56 (.70) | 3.53 (.70) | |
| Self-esteem | 3.11 (1.10) | 3.97 (.68) | 3.07 (1.27) | 3.56 (1.02) | 3.25 (1.30) | 3.76 (.70) | 3.56 (1.18) | 3.77 (1.13) | |
| Unpleasant Emotions | 2.32 (.71) | 1.91 (.55) | 2.29 (.64) | 2.04 (.67) | 2.35 (.77 | 1.87 (.60) | 2.07 (.669 | 2.09 (.63) | |
| Maladaptive Coping | 1.95 (.46) | 1.69 (.30) | 1.95 (.44) | 1.78 (.30) | 2.00 (.48) | 1.73 (.46) | 1.80 (.41) | 1.82 (.50) | |
| **Health Outcomes** |  |  |  |  |  |  |  |  |  |
| Physical Health | 3.18 (.92) | 3.48 (.87) | 3.18 (1.04) | 3.38 (.92) | 2.89 (1.01 | 3.24 (1.09) | 3.62 (1.03) | 3.62 (.85) | |
| Sleep Problems | 5.55 (3.93) | 2.97 (2.26) | 5.91 (4.34) | 4.21 (3.62) | 4.72 (3.51) | 2.95 (3.22) | 4.13 (3.91) | 3.70 (3.50) | |

**Table C.** Mann-Whitney Test Results - Comparison of Change Scores from Time 1 to Time 2

|  | | | |
| --- | --- | --- | --- |
|  | SKY vs. Control | MBSR vs. Control | EI vs. Control |
|  | *p* | *p* | p |
| **Mental Health** |  |  |  |
| Burnout | .001 | .019 | .005 |
| Stress | .000 | .017 | .006 |
| MASQ Distress | .024 | .139 | .111 |
| MASQ Depression | .000 | .023 | .026 |
| MASQ Anxiety | .048 | .112 | .091 |
| Mental Health | .000 | .396 | .000 |
| **Psychological Thriving** |  |  |  |
| Life satisfaction | .014 | .055 | .003 |
| Ryff Autonomy | .145 | .063 | .846 |
| Ryff Environment | .000 | .144 | .002 |
| Ryff Growth | .027 | .161 | .016 |
| Ryff Relations | .054 | .233 | .247 |
| Ryff Purpose | .053 | .824 | .292 |
| Ryff Self-acceptance | .001 | .037 | .026 |
| Pleasant Emotions | .000 | .000 | .001 |
| Social Connectedness | .027 | .137 | .000 |
| Gratitude | .145 | .426 | .047 |
| Self-Compassion | .005 | .117 | .096 |
| Mindfulness | .000 | .006 | .000 |
| Adaptive Coping | .021 | .023 | .022 |
| Optimism | .138 | .018 | .239 |
| Self-esteem | .004 | .404 | .119 |
| Unpleasant Emotions | .000 | 000 | .035 |
| Maladaptive Coping | .007 | .007 | .006 |
| **Health Outcomes** |  |  |  |
| Physical Health | .054 | .136 | .080 |
| Sleep Problems | .002 | .227 | .194 |

**Table D.** Paired-Samples T-Test from Time 1 to Time 2

|  | SKY | | MBSR | | EI | | Control | |
| --- | --- | --- | --- | --- | --- | --- | --- | --- |
|  | t | p | t | p | t | p | t | p |
| **Mental Health** |  |  |  |  |  |  |  |  |
| Burnout | 1.49 | .147 | .172 | .865 | .591 | .561 | -3.58 | .001 |
| Stress | 4.21 | .000 | 1.71 | .098 | 1.47 | .157 | -1.73 | .091 |
| MASQ Distress | 4.04 | .000 | 2.61 | .013 | 1.70 | .106 | 2.17 | .035 |
| MASQ Depression | 3.48 | .002 | 1.82 | .077 | 1.50 | .148 | -1.45 | .155 |
| MASQ Anxiety | 2.83 | .009 | 2.15 | .039 | 2.22 | .038 | .93 | .359 |
| Mental Health | -4.74 | .000 | -2.85 | .778 | -4.20 | .000 | .41 | .688 |
| **Psychological Thriving** |  |  |  |  |  |  |  |  |
| Life satisfaction | -4.04 | .000 | -3.62 | .001 | -3.82 | .001 | -2.08 | .043 |
| Ryff Autonomy | -1.76 | .090 | -2.11 | .043 | -.233 | .818 | .22 | .824 |
| Ryff Environment | -3.75 | .001 | -1.31 | .199 | -3.54 | .022 | .76 | .452 |
| Ryff Growth | -1.79 | .084 | -1.30 | .203 | -2.63 | .016 | .21 | .832 |
| Ryff Relations | -2.83 | .009 | -2.21 | .034 | -2.23 | .038 | -.63 | .529 |
| Ryff Purpose | .365 | .718 | 1.14 | .263 | -.904 | .377 | 1.80 | .079 |
| Ryff Self-acceptance | -3.22 | .003 | -1.76 | .088 | -1.96 | .064 | 1.81 | .078 |
| Pleasant Emotions | -2.43 | .022 | -2.88 | .007 | -.49 | .633 | 3.01 | .004 |
| Social Connectedness | -3.77 | .001 | -1.65 | .109 | -3.54 | .002 | -.31 | .756 |
| Gratitude | -2.53 | .018 | -1.44 | .160 | -2.57 | .018 | -.59 | .559 |
| Self-Compassion | -4.04 | .000 | -2.54 | .016 | -2.40 | .027 | -2.38 | .022 |
| Mindfulness | -3.80 | .001 | -3.14 | .004 | -4.86 | .000 | -.19 | .850 |
| Adaptive Coping | -1.96 | .060 | -1.76 | .087 | -1.43 | .169 | 1.04 | .306 |
| Optimism | -1.76 | .090 | -2.42 | .021 | -1.62 | .121 | .103 | .918 |
| Self-esteem | -5.19 | .000 | -2.52 | .017 | -2.95 | .008 | -1.66 | .103 |
| Unpleasant Emotions | 3.27 | .003 | 1.36 | .183 | 1.69 | .107 | -.832 | .409 |
| Maladaptive Coping | 2.92 | .007 | 1.73 | .092 | 2.15 | .044 | -.94 | .352 |
| **Health Outcomes** |  |  |  |  |  |  |  |  |
| Physical Health | -2.12 | .043 | -1.56 | .128 | -2.02 | .056 | .00 | 1.000 |
| Sleep Problems | 4.16 | .000 | 1.15 | .258 | 1.36 | .189 | .88 | .385 |

Note. P = significance, 2-tailed

**Table E:** Attrition analysis from T1 to T2

| **Outcome Variable** | Means of Participants who Dropped Out Between T1 and T2  (n = 58) | Means of Participants who Stayed from T1 to T2 (n = 131) | *p* |
| --- | --- | --- | --- |
| **Mental Health** |  |  |  |
| Burnout | 2.52 (.80) | 2.29 (.73) | 0.088 |
| Stress | 3.03 (.63) | 2.78 (.59) | 0.013 |
| MASQ Distress | 2.77 (.82) | 2.55 (.76) | 0.119 |
| MASQ Depression | 2.93 (.77) | 2.93 (.77) | 0.965 |
| MASQ Anxiety | 1.87 (.63) | 1.77 (.60) | 0.404 |
| Mental Health | 2.78 (1.09) | 3.08 (1.15) | 0.118 |
| **Psychological Thriving**  Life satisfaction | 4.66 (1.25) | 4.90 (1.22) | 0.272 |
| Ryff Autonomy | 5.64 (1.41) | 4.88 (1.28) | 0.200 |
| Ryff Environment | 4.21 (1.23 | 4.54 (1.08) | 0.112 |
| Ryff Growth | 6.46 (.62) | 6.27 (.74) | 0.107 |
| Ryff Relations | 5.25 (1.30) | 5.18 (1.38) | 0.821 |
| Ryff Purpose | 5.69 (.89) | 5.56 (1.01) | 0.576 |
| Ryff Self-acceptance | 5.21 (1.32) | 5.50 (1.16) | 0.191 |
| Pleasant Emotions | 3.06 (.70) | 2.98 (.66) | 0.401 |
| Social Connectedness | 4.10 (.93) | 4.17 (.92) | 0.811 |
| Gratitude | 5.93 (1.03) | 5.93 (.98) | 0.987 |
| Self-Compassion | 2.60 (.55) | 2.84 (.78) | 0.056 |
| Mindfulness | 3.12 (.55) | 3.19 (.53) | 0.473 |
| Adaptive Coping | 2.75 (.53) | 2.71 (.57) | 0.530 |
| Optimism | 3.27 (.87) | 3.37 (.74) | 0.464 |
| Self-esteem | 3.07 (1.30) | 3.34 (1.18) | 0.238 |
| Unpleasant Emotions | 2.41 (.76) | 2.17 (.66) | 0.045 |
| Maladaptive Coping | 2.03 (.52) | 1.87 (.40) | 0.036 |
| **Health Outcomes**  Physical Health | 3.12 (1.04) | 3.31 (1.02) | 0.303 |
| Sleep Problems | 5.67 (4.14) | 4.83 (3.94) | 0.143 |

To examine possible self-selection biases, we compared the mean scores at T1 for the group that completed the study (*N* = 131) and the group that dropped out after T1 (*N* = 58) using a nonparametric Mann-Whitney-U-test. Means at T1 for all outcomes are presented in Table E. The groups of drop-outs after T1 and study completers differed in three variables significantly and with small to moderate effect sizes, namely in stress (*d* = .41), unpleasant emotions (*d* = .34), maladaptive coping (*d* = .46). All other outcome variables were similar between the two groups. This suggests that stress may have played a role in the motives to drop out of the treatment and study. Since the students who later dropped out were removed from all other analyses, the difference between drop-outs and completers should only affect the generalizability of the findings, but not the findings of treatment effects themselves.

**Comparisons among intervention groups for the outcomes for which multiple interventions showed significant differences to the control group:**

For those outcomes for which two or more intervention groups showed significantly (p ≤ .05) different scores than the control group at T2, we examined whether there were any significant differences between the interventions that differed from the control group. For that purpose, we used the same autoregressive models as before, with different dummy variables. Each of the intervention groups was used as the reference group in the dummy variable creation once, so that all pairwise differences between all groups were examined (results see tables F-N below). There were no significant differences between any of the intervention groups in the outcomes for which two or more intervention groups showed significantly different scores than the control group at T2. Below is a list of these outcomes and the two or more intervention groups that showed significantly different scores than the control group at T2 for these outcomes.

- Mental health: SKY vs. EI: No significant difference
- Adaptive coping:
  - EI vs. SKY: No significant difference
  - EI vs. MBSR: no significant difference
  - MBSR vs. SKY: no significant difference
- Mindfulness:
  - EI vs. SKY: no significant difference
  - EI vs. MBSR: no significant difference
  - MBSR vs. SKY: no significant difference
- Gratitude: SKY vs. EI: no significant difference
- Connectedness: SKY vs. EI: no significant difference
- Pleasant emotions: SKY vs. MBSR: no significant difference
- Self-acceptance: MBSR vs. EI: no significant difference
- Autonomy (RYFF): SKY vs. MBSR: no significant difference
- Environment-related well-being (RYFF): SKY vs. EI: no significant difference
- Growth (RYFF): SKY vs. EI: no significant difference
- Self-acceptance (RYFF): SKY vs. EI: no significant difference
- Life satisfaction: SKY vs. EI: no significant difference
- Anxiety (MASQ): SKY vs. EI: no significant difference
- Negative affect (PANAS): SKY vs. EI: no significant difference
- Maladaptive coping: SKY vs. EI: no significant difference
- Burnout: SKY vs. MBSR: no significant difference

**Table F:** From T1 to T2: Comparing all groups to the SKY intervention: Regressions for mental health outcomes regressed on groups

|  | Outcome Time 1 | | | |  | Outcome Time 2 | | | |
| --- | --- | --- | --- | --- | --- | --- | --- | --- | --- |
| Predictors | *B* | *SE(B)* | β | *p (2-tailed)* |  | *B* | *SE(B)* | β | *p (2-tailed)* |
| **Burnout T1** |  |  |  |  |  | 0.18* | 0.09 | .18 | .05 |
| MBSR vs. SKY ^a^ | 0.01 | 0.14 | .01 | .95 |  | 0.09 | 0.17 | .05 | .63 |
| EI vs. SKY ^b^ | 0.20 | 0.18 | .11 | .25 |  | 0.12 | 0.21 | .06 | .56 |
| Control vs. SKY ^c^ | -0.32* | 0.14 | -.19 | .03 |  | 0.41* | 0.17 | .25 | .02 |
| *R^2^* | 0.06 |  |  | .08 |  | 0.06 |  |  | .16 |
| **Stress T1** |  |  |  |  |  | 0.36* | 0.09 | .35 | .00 |
| MBSR vs. SKY ^a^ | -0.02 | 0.13 | -.01 | .89 |  | 0.26 | 0.14 | .19 | .07 |
| EI vs. SKY ^b^ | 0.07 | 0.14 | .05 | .60 |  | 0.27 | 0.17 | .17 | .11 |
| Control vs. SKY ^c^ | -0.30* | 0.13 | -.22 | .02 |  | 0.47* | 0.11 | .34 | .00 |
| *R^2^* | 0.06 |  |  | .08 |  | 0.17* |  |  | .01 |
| **Distress T1** |  |  |  |  |  | 0.34* | 0.07 | .41 | .00 |
| MBSR vs. SKY ^a^ | -0.09 | 0.16 | -.05 | .58 |  | 0.08 | 0.13 | .06 | .55 |
| EI vs. SKY ^b^ | 0.03 | 0.19 | .02 | .88 |  | 0.16 | 0.17 | .10 | .35 |
| Control vs. SKY ^c^ | -0.17 | 0.17 | -.10 | .30 |  | 0.24* | 0.12 | .17 | .04 |
| *R^2^* | 0.01 |  |  | .51 |  | 0.18* |  |  | .00 |
| **Depression T1** |  |  |  |  |  | 0.47* | 0.08 | .48 | .00 |
| MBSR vs. SKY ^a^ | 0.14 | 0.15 | .08 | .35 |  | 0.37* | 0.17 | .23 | .03 |
| EI vs. SKY ^b^ | 0.01 | 0.16 | .01 | .94 |  | 0.32 | 0.20 | .17 | .11 |
| Control vs. SKY ^c^ | -0.29 | 0.15 | -.17 | .06 |  | 0.50* | 0.14 | .31 | .00 |
| *R^2^* | 0.05 |  |  | .14 |  | 0.27* |  |  | .00 |
| **Anxiety T1** |  |  |  |  |  | 0.51* | 0.06 | .63 | .00 |
| MBSR vs. SKY ^a^ | -0.06 | 0.13 | -.05 | .63 |  | 0.01 | 0.09 | .01 | .89 |
| EI vs. SKY ^b^ | -0.09 | 0.15 | -.06 | .56 |  | -0.03 | 0.10 | -.02 | .80 |
| Control vs. SKY ^c^ | -0.20 | 0.13 | -.15 | .12 |  | 0.14 | 0.09 | .12 | .11 |
| *R^2^* | 0.02 |  |  | .38 |  | 0.39* |  |  | .00 |
| **Mental Health T1** |  |  |  |  |  | 0.63* | 0.06 | .64 | .00 |
| MBSR vs. SKY ^a^ | 0.18 | 0.21 | .07 | .41 |  | 0.78* | 0.23 | .31 | .00 |
| EI vs. SKY ^b^ | 0.31 | 0.26 | .11 | .23 |  | 0.19 | 0.21 | .07 | .36 |
| Control vs. SKY ^c^ | -0.41 | 0.23 | -.16 | .07 |  | 0.66* | 0.17 | .26 | .00 |
| *R^2^* | 0.06 |  |  | .09 |  | 0.47* |  |  | .00 |

*Notes*. * : *p(one-tailed)* ≤ .05; ** : *p(one-tailed)* ≤ .01; ***: *p(one-tailed)* ≤ .000; ^a^ coded 1 = MBSR treatment group, 0 = all other groups; ^b^ coded 1 = EI treatment group, 0 = all other groups; ^c^ coded 1 = Control treatment group, 0 = all other groups; This table uses 2-tailed p-values because we had no directed hypotheses for the comparisons among the three intervention groups.

**Table G:** Comparing all groups to the SKY intervention: From T1 to T2: Regressions for well-being outcomes regressed on groups

|  | Outcome Time 1 | | | |  | Outcome Time 2 | | | |
| --- | --- | --- | --- | --- | --- | --- | --- | --- | --- |
| Predictors | *B* | *SE(B)* | β | *p (2-tailed)* |  | *B* | *SE(B)* | β | *p (2-tailed)* |
| **Life Satisfaction T1** |  |  |  |  |  | 0.57* | 0.06 | .71 | .00 |
| MBSR vs. SKY ^a^ | 0.10 | 0.24 | .04 | .68 |  | -0.24 | 0.20 | -.11 | .24 |
| EI vs. SKY ^b^ | 0.09 | 0.29 | .03 | .76 |  | 0.03 | 0.20 | .01 | .90 |
| Control vs. SKY ^c^ | 0.70* | 0.24 | .26 | .00 |  | -0.35* | 0.17 | -.16 | .04 |
| *R^2^* | 0.06 |  |  | .06 |  | 0.48* |  |  | .00 |
| **Ryff Autonomy T1** |  |  |  |  |  | 0.59* | 0.05 | .69 | .00 |
| MBSR vs. SKY ^a^ | -0.10 | 0.28 | -.03 | .722 |  | 0.00 | 0.18 | .00 | .99 |
| EI vs. SKY ^b^ | -0.02 | 0.31 | -.01 | .942 |  | -0.33 | 0.25 | -.11 | .20 |
| Control vs. SKY ^c^ | -0.05 | 0.26 | -.02 | .835 |  | -0.38* | 0.18 | -.15 | .03 |
| *R^2^* | 0.00 |  |  | .851 |  | 0.50* |  |  | .00 |
| **Ryff Environment T1** |  |  |  |  |  | 0.49* | 0.08 | .51 | .00 |
| MBSR vs. SKY ^a^ | -0.09 | 0.23 | -.04 | .688 |  | -0.48 | 0.25 | -.21 | .06 |
| EI vs. SKY ^b^ | -0.26 | 0.27 | -.09 | .330 |  | -0.28 | 0.28 | -.10 | .32 |
| Control vs. SKY ^c^ | 0.51 | 0.23 | .20 | .027 |  | -0.68* | 0.21 | -.29 | .00 |
| *R^2^* | 0.07 |  |  | .048 |  | 0.28* |  |  | .00 |
| **Ryff Growth T1** |  |  |  |  |  | 0.43* | 0.07 | .50 | .00 |
| MBSR vs. SKY ^a^ | 0.01 | 0.14 | .01 | .930 |  | -0.12 | 0.14 | -.09 | .41 |
| EI vs. SKY ^b^ | -0.07 | 0.17 | -.04 | .700 |  | 0.06 | 0.13 | .04 | .63 |
| Control vs. SKY ^c^ | 0.02 | 0.14 | .01 | .895 |  | -0.22 | 0.13 | -.17 | .09 |
| *R^2^* | 0.00 |  |  | .785 |  | 0.27 |  |  | .00 |
| **Ryff Relations T1** |  |  |  |  |  | 0.64* | 0.05 | .74 | .00 |
| MBSR vs. SKY ^a^ | -0.34 | 0.28 | -.11 | .231 |  | -0.34 | 0.22 | -.13 | .12 |
| EI vs. SKY ^b^ | -0.30 | 0.33 | -.09 | .361 |  | -0.28 | 0.21 | -.09 | .19 |
| Control vs. SKY ^c^ | 0.11 | 0.27 | .04 | .680 |  | -0.42* | 0.18 | -.16 | .02 |
| *R^2^* | 0.02 |  |  | .294 |  | 0.57* |  |  | .00 |
| **Ryff Purpose T1** |  |  |  |  |  | 0.57* | 0.08 | .57 | .00 |
| MBSR vs. SKY ^a^ | -0.04 | 0.20 | -.02 | .858 |  | -0.18 | 0.21 | -.08 | .41 |
| EI vs. SKY ^b^ | -0.33 | 0.24 | -.13 | .176 |  | 0.07 | 0.23 | .03 | .77 |
| Control vs. SKY ^c^ | 0.05 | 0.19 | .02 | .811 |  | -0.05 | 0.18 | -.03 | .77 |
| *R^2^* | 0.02 |  |  | .390 |  | 0.33* |  |  | .00 |
| **Ryff Self-Acceptance** |  |  |  |  |  | 0.44* | 0.05 | .56 | .00 |
| MBSR vs. SKY ^a^ | -0.08 | 0.24 | -.03 | .733 |  | -0.29 | 0.19 | -.14 | .13 |
| EI vs. SKY ^b^ | -0.04 | 0.30 | -.01 | .907 |  | -0.12 | 0.18 | -.05 | .50 |
| Control vs. SKY ^c^ | 0.36 | 0.23 | .14 | .112 |  | -0.57* | 0.17 | -.27 | .00 |
| *R^2^* | 0.02 |  |  | .235 |  | 0.33* |  |  | .00 |
| **Pleasant Emotions T1** |  |  |  |  |  | 0.56* | 0.07 | .54 | .00 |
| MBSR vs. SKY ^a^ | -0.21 | 0.14 | -.13 | .14 |  | -0.10 | 0.17 | -.06 | .55 |
| EI vs. SKY ^b^ | -0.10 | 0.16 | -.06 | .51 |  | -0.35* | 0.16 | -.19 | .03 |
| Control vs. SKY ^c^ | 0.10 | 0.14 | .07 | .48 |  | -0.51* | 0.15 | -.32 | .00 |
| *R^2^* | 0.03 |  |  | .22 |  | 0.35* |  |  | .00 |
| **Connectedness T1** |  |  |  |  |  | 0.65* | 0.06 | .75 | .00 |
| MBSR vs. SKY ^a^ | -0.04 | 0.19 | -.02 | .85 |  | -0.35* | 0.14 | -.20 | .01 |
| EI vs. SKY ^b^ | -0.21 | 0.21 | -.09 | .32 |  | -0.13 | 0.14 | -.06 | .35 |
| Control vs. SKY ^c^ | 0.22 | 0.20 | .11 | .27 |  | -0.39* | 0.11 | -.22 | .00 |
| *R^2^* | 0.03 |  |  | .24 |  | 0.57* |  |  | .00 |
| **Gratitude T1** |  |  |  |  |  | 0.57* | 0.06 | .69 | .00 |
| MBSR vs. SKY ^a^ | -0.24 | 0.21 | -.11 | .25 |  | -0.29* | 0.15 | -.16 | .05 |
| EI vs. SKY ^b^ | -0.16 | 0.24 | -.06 | .52 |  | 0.00 | 0.16 | .00 | 1.00 |
| Control vs. SKY ^c^ | 0.06 | 0.20 | .03 | .77 |  | -0.27* | 0.13 | -.15 | .03 |
| *R^2^* | 0.02 |  |  | .36 |  | 0.51* |  |  | .00 |
| **Self-Compassion T1** |  |  |  |  |  | 0.47* | 0.08 | .52 | .00 |
| MBSR vs. SKY ^a^ | -0.03 | 0.16 | -.02 | .84 |  | -0.23 | 0.17 | -.15 | .17 |
| EI vs. SKY ^b^ | 0.05 | 0.19 | .03 | .78 |  | -0.10 | 0.19 | -.05 | .61 |
| Control vs. SKY ^c^ | 0.26 | 0.17 | .14 | .12 |  | -0.35* | 0.14 | -.22 | .01 |
| *R^2^* | 0.02 |  |  | .28 |  | 0.29* |  |  | .00 |
| **Mindfulness T1** |  |  |  |  |  | 0.58* | 0.07 | .62 | .00 |
| MBSR vs. SKY ^a^ | -0.08 | 0.11 | -.07 | .45 |  | -0.12 | 0.11 | -.11 | .27 |
| EI vs. SKY ^b^ | -0.24* | 0.11 | -.18 | .04 |  | 0.05 | 0.11 | .04 | .65 |
| Control vs. SKY ^c^ | 0.09 | 0.11 | .08 | .40 |  | -0.31* | 0.09 | -.28 | .00 |
| *R^2^* | 0.05 |  |  | .09 |  | 0.40* |  |  | .00 |
| **Adaptive Coping T1** |  |  |  |  |  | 0.55* | 0.07 | .53 | .00 |
| MBSR vs. SKY ^a^ | 0.01 | 0.11 | .01 | .91 |  | -0.05 | 0.13 | -.04 | .69 |
| EI vs. SKY ^b^ | 0.04 | 0.13 | .03 | .77 |  | -0.01 | 0.13 | -.01 | .91 |
| Control vs. SKY ^c^ | 0.02 | 0.12 | .02 | .85 |  | -0.26* | 0.12 | -.21 | .03 |
| *R^2^* | 0.00 |  |  | .88 |  | 0.32* |  |  | .00 |
| **Negative affect T1** |  |  |  |  |  | 0.38* | 0.09 | .42 | .00 |
| MBSR vs. SKY ^a^ | -0.04 | 0.14 | -.02 | .79 |  | 0.15 | 0.15 | .11 | .31 |
| EI vs. SKY ^b^ | 0.03 | 0.16 | .02 | .87 |  | 0.02 | 0.16 | .01 | .89 |
| Control vs. SKY ^c^ | -0.26 | 0.14 | -.17 | .07 |  | 0.29* | 0.12 | .21 | .01 |
| *R^2^* | 0.03 |  |  | .25 |  | 0.19* |  |  | .01 |
| **Maladaptive Coping T1** |  |  |  |  |  | 0.47* | 0.10 | .50 | .00 |
| MBSR vs. SKY ^a^ | 0.01 | 0.09 | .01 | .94 |  | 0.07 | 0.07 | .08 | .30 |
| EI vs. SKY ^b^ | 0.06 | 0.11 | .05 | .59 |  | -0.02 | 0.11 | -.02 | .87 |
| Control vs. SKY ^c^ | -0.14 | 0.09 | -.15 | .10 |  | 0.18* | 0.08 | .20 | .02 |
| *R^2^* | .03 |  |  | .21 |  | .26* |  |  | .00 |

*Notes*. * : *p(one-tailed)* ≤ .05; ** : *p(one-tailed)* ≤ .01; ***: *p(one-tailed)* ≤ .000; ^a^ coded 1 = MBSR treatment group, 0 = all other groups; ^b^ coded 1 = EI treatment group, 0 = all other groups; ^c^ coded 1 = Control treatment group, 0 = all other groups; This table uses 2-tailed p-values because we had no directed hypotheses for the comparisons among the three intervention groups.

**Table H:** Comparing all groups to the SKY intervention: From T1 to T2: Regressions for health well-being outcomes regressed on groups

|  | Time 1 | | | |  | Time 2 | | | |
| --- | --- | --- | --- | --- | --- | --- | --- | --- | --- |
|  | *B* | *SE(B)* | β | *p (2-tailed)* |  | *B* | *SE(B)* | β | *p (2-tailed)* |
| **Physical Health T1** |  |  |  |  |  | 0.68* | 0.05 | .77 | .00 |
| MBSR vs. SKY ^a^ | 0.00 | 0.20 | .00 | 1.00 |  | 0.08 | 0.16 | .04 | .62 |
| EI vs. SKY ^b^ | 0.29 | 0.22 | .11 | .17 |  | 0.10 | 0.18 | .04 | .56 |
| Control vs. SKY ^c^ | -0.45* | 0.20 | -.20 | .02 |  | 0.15 | 0.14 | .07 | .29 |
| *R^2^* | 0.07 |  |  | .06 |  | 0.58* |  |  | .00 |
| **Sleep Problems T1** |  |  |  |  |  | 0.27* | 0.08 | .33 | .00 |
| MBSR vs. SKY ^a^ | 0.36 | 0.82 | .04 | .66 |  | 1.43 | 0.75 | .20 | .06 |
| EI vs. SKY ^b^ | -0.82 | 0.82 | -.08 | .32 |  | 0.50 | 0.78 | .06 | .52 |
| Control vs. SKY ^c^ | -1.42 | 0.79 | -.16 | .07 |  | 1.21 | 0.64 | .17 | .06 |
| *R^2^* | 0.03 |  |  | .20 |  | 0.14* |  |  | .02 |

*Notes*. * : *p(two-tailed)* ≤ .05; ^a^ coded 1 = MBSR treatment group, 0 = all other groups; ^b^ coded 1 = EI treatment group, 0 = all other groups; ^c^ coded 1 = Control treatment group, 0 = all other groups; This table uses 2-tailed p-values because we had no directed hypotheses for the comparisons among the three intervention groups.

**Table I:** From T1 to T2: Comparing all groups to the EI intervention: Comparing all groups to the EI intervention: Regressions for mental health outcomes regressed on groups

|  | Outcome Time 1 | | | | | |  | Outcome Time 2 | | | | | | | |
| --- | --- | --- | --- | --- | --- | --- | --- | --- | --- | --- | --- | --- | --- | --- | --- |
| Predictors | *B* | *SE(B)* | | β | | *p (2-tailed)* |  | *B* | | *SE(B)* | | β | | *p (2-tailed)* | |
| **Burnout T1** |  |  | |  | |  |  | 0.18* | | 0.09 | | .18 | | .05 | |
| SKY vs. EI ^a^ | -0.20 | 0.18 | | -.11 | | .25 |  | -0.12 | | 0.21 | | -.07 | | .56 | |
| MBSR vs. EI ^b^ | -0.19 | 0.17 | | -.12 | | .25 |  | -0.04 | | 0.21 | | -.02 | | .86 | |
| Control vs. EI ^c^ | -0.52* | 0.17 | | -.31 | | .00 |  | 0.29 | | 0.21 | | .17 | | .18 | |
| *R^2^* | 0.06 |  | |  | | .08 |  | 0.06 | |  | |  | | .16 | |
| **Stress T1** |  |  | |  | |  |  | 0.36* | | 0.09 | | .35 | | .00 | |
| SKY vs. EI ^a^ | -0.07 | 0.14 | | -.05 | | .60 |  | -0.27 | | 0.17 | | -.19 | | .11 | |
| MBSR vs. EI ^b^ | -0.09 | 0.13 | | -.07 | | .46 |  | -0.01 | | 0.19 | | -.01 | | .95 | |
| Control vs. EI ^c^ | -0.37* | 0.13 | | -.27 | | .00 |  | 0.20 | | 0.17 | | .14 | | .25 | |
| *R^2^* | 0.06 |  | |  | | .08 |  | 0.17* | |  | |  | | .01 | |
| **Distress T1** |  |  | |  | |  |  | 0.34* | | 0.07 | | .41 | | .00 | |
| SKY vs. EI ^a^ | -0.03 | 0.19 | | -.02 | | .88 |  | -0.16 | | 0.17 | | -.11 | | .35 | |
| MBSR vs. EI ^b^ | -0.12 | 0.17 | | -.07 | | .49 |  | -0.08 | | 0.19 | | -.06 | | .66 | |
| Control vs. EI ^c^ | -0.20 | 0.18 | | -.12 | | .26 |  | 0.08 | | 0.18 | | .05 | | .66 | |
| *R^2^* | 0.01 |  | |  | | .51 |  | 0.18* | |  | |  | | .00 | |
| **Depression T1** |  |  | |  | |  |  | 0.47* | | 0.08 | | .48 | | .00 | |
| SKY vs. EI ^a^ | -0.01 | 0.16 | | -.01 | | .94 |  | -0.32 | | 0.20 | | -.18 | | .11 | |
| MBSR vs. EI ^b^ | 0.13 | 0.16 | | .08 | | .42 |  | 0.05 | | 0.20 | | .03 | | .78 | |
| Control vs. EI ^c^ | -0.30 | 0.16 | | -.18 | | .06 |  | 0.19 | | 0.17 | | .12 | | .28 | |
| *R^2^* | 0.05 |  | |  | | .14 |  | 0.27* | |  | |  | | .00 | |
| **Anxiety T1** |  |  | |  | |  |  | 0.51* | | 0.06 | | .63 | | .00 | |
| SKY vs. EI ^a^ | 0.09 | 0.15 | | .06 | | .56 |  | 0.03 | | 0.10 | | .02 | | .80 | |
| MBSR vs. EI ^b^ | 0.03 | 0.13 | | .02 | | .85 |  | 0.04 | | 0.10 | | .04 | | .72 | |
| Control vs. EI ^c^ | -0.11 | 0.13 | | -.08 | | .41 |  | 0.16 | | 0.10 | | .15 | | .10 | |
| *R^2^* | 0.02 |  | |  | | .38 |  | 0.39* | |  | |  | | .00 | |
| **Mental Health T1** |  | |  | |  |  |  | 0.63* | 0.06 | | .64 | | .00 | |  |
| SKY vs. EI ^a^ | -0.31 | | 0.26 | | -.12 | .23 |  | -0.19 | 0.21 | | -.07 | | .36 | |  |
| MBSR vs. EI ^b^ | -0.13 | | 0.24 | | -.05 | .58 |  | 0.59* | 0.24 | | .24 | | .01 | |  |
| Control vs. EI ^c^ | -0.72* | | 0.25 | | -.29 | .00 |  | 0.47* | 0.19 | | .19 | | .02 | |  |
| *R^2^* | 0.06 | |  | |  | .09 |  | 0.47* |  | |  | | .00 | |  |

*Notes*. * : *p(two-tailed)* ≤ .05; ^a^ coded 1 = SKY treatment group, 0 = all other groups; ^b^ coded 1 = MBSR treatment group, 0 = all other groups; ^c^ coded 1 = Control group, 0 = all other groups; This table uses 2-tailed p-values because we had no directed hypotheses for the comparisons among the three intervention groups.

**Table J:** Comparing all groups to the EI intervention: From T1 to T2: Regressions for well-being outcomes regressed on groups

|  | Outcome Time 1 | | | | |  | | Outcome Time 2 | | | | |
| --- | --- | --- | --- | --- | --- | --- | --- | --- | --- | --- | --- | --- |
| Predictors | *B* | *SE(B)* | β | | *p (2-tailed)* |  | | *B* | | *SE(B)* | β | *p (2-tailed)* |
| **Life Satisfaction T1** |  |  |  | |  |  | | 0.57* | | 0.06 | .71 | .00 |
| SKY vs. EI ^a^ | -0.09 | 0.29 | -.03 | | .76 |  | | -0.03 | | 0.20 | -.01 | .90 |
| MBSR vs. EI ^b^ | 0.01 | 0.28 | .00 | | .98 |  | | -0.26 | | 0.19 | -.12 | .18 |
| Control vs. EI ^c^ | 0.61* | 0.27 | .23 | | .03 |  | | -0.38* | | 0.18 | -.17 | .03 |
| *R^2^* | 0.06 |  |  | | .06 |  | | 0.48* | |  |  | .00 |
| **Ryff Autonomy T1** |  |  |  | |  |  | | 0.59* | | 0.05 | .69 | .00 |
| SKY vs. EI ^a^ | 0.02 | 0.31 | .01 | | .94 |  | | 0.33 | | 0.25 | .12 | .20 |
| MBSR vs. EI ^b^ | -0.08 | 0.30 | -.03 | | .80 |  | | 0.33 | | 0.25 | .13 | .19 |
| Control vs. EI ^c^ | -0.03 | 0.28 | -.01 | | .91 |  | | -0.05 | | 0.25 | -.02 | .83 |
| *R^2^* | 0.00 |  |  | | .85 |  | | 0.50* | |  |  | .00 |
| **Ryff Environment T1** |  |  |  | |  |  | | 0.49* | | 0.08 | .51 | .00 |
| SKY vs. EI ^a^ | 0.26 | 0.27 | .10 | | .33 |  | | 0.28 | | 0.28 | .11 | .32 |
| MBSR vs. EI ^b^ | 0.17 | 0.24 | .07 | | .48 |  | | -0.21 | | 0.27 | -.09 | .44 |
| Control vs. EI ^c^ | 0.77* | 0.24 | .31 | | .00 |  | | -0.40 | | 0.23 | -.17 | .08 |
| *R^2^* | 0.07* |  |  | | .05 |  | | 0.28* | |  |  | .00 |
| **Ryff Growth T1** |  |  |  | |  |  | | 0.43* | | 0.07 | .50 | .00 |
| SKY vs. EI ^a^ | 0.07 | 0.17 | .04 | | .70 |  | | -0.06 | | 0.13 | -.04 | .63 |
| MBSR vs. EI ^b^ | 0.08 | 0.16 | .05 | | .63 |  | | -0.18 | | 0.12 | -.13 | .14 |
| Control vs. EI ^c^ | 0.08 | 0.16 | .05 | | .61 |  | | -0.28 | | 0.11 | -.21 | .01 |
| *R^2^* | 0.00 |  |  | | .79 |  | | 0.27* | |  |  | .00 |
| **Ryff Relations T1** |  |  |  | |  |  | | 0.64* | | 0.05 | .74 | .00 |
| SKY vs. EI ^a^ | 0.30 | 0.33 | .09 | | .36 |  | | 0.28 | | 0.21 | .10 | .19 |
| MBSR vs. EI ^b^ | -0.04 | 0.30 | -.01 | | .89 |  | | -0.06 | | 0.21 | -.03 | .77 |
| Control vs. EI ^c^ | 0.41 | 0.29 | .14 | | .16 |  | | -0.15 | | 0.17 | -.06 | .40 |
| *R^2^* | 0.02 |  |  | | .29 |  | | 0.57* | |  |  | .00 |
| **Ryff Purpose T1** |  |  |  | |  |  | | 0.57* | | 0.08 | .57 | .00 |
| SKY vs. EI ^a^ | 0.33 | 0.24 | .14 | | .18 |  | | -0.07 | | 0.23 | -.03 | .77 |
| MBSR vs. EI ^b^ | 0.29 | 0.23 | .14 | | .21 |  | | -0.25 | | 0.24 | -.11 | .30 |
| Control vs. EI ^c^ | 0.37 | 0.22 | .17 | | .09 |  | | -0.12 | | 0.21 | -.06 | .55 |
| *R^2^* | 0.02 |  |  | | .39 |  | | 0.33* | |  |  | .00 |
| **Ryff Self-Acceptance** |  |  |  | |  |  | | 0.44* | | 0.05 | .56 | .00 |
| SKY vs. EI ^a^ | 0.04 | 0.30 | .01 | | .91 |  | | 0.12 | | 0.18 | .05 | .50 |
| MBSR vs. EI ^b^ | -0.05 | 0.29 | -.02 | | .88 |  | | -0.17 | | 0.20 | -.08 | .40 |
| Control vs. EI ^c^ | 0.40 | 0.28 | .15 | | .16 |  | | -0.45* | | 0.18 | -.21 | .02 |
| *R^2^* | 0.02 |  |  | | .24 |  | | 0.33 | |  |  | .00 |
| **Pleasant Emotions T1** |  |  |  | |  |  | | 0.56* | | 0.07 | .54 | .00 |
| SKY vs. EI ^a^ | 0.10 | 0.16 | .06 | | .51 |  | | 0.35* | | 0.16 | .21 | .03 |
| MBSR vs. EI ^b^ | -0.10 | 0.14 | -.07 | | .47 |  | | 0.25 | | 0.15 | .16 | .10 |
| Control vs. EI ^c^ | 0.20 | 0.15 | .13 | | .17 |  | | -0.16 | | 0.13 | -.10 | .21 |
| *R^2^* | 0.03 |  |  | | .22 |  | | 0.35* | |  |  | .00 |
| **Connectedness T1** |  |  |  | |  |  | | 0.65* | | 0.06 | .75 | .00 |
| SKY vs. EI ^a^ | 0.21 | 0.21 | .10 | | .32 |  | | 0.13 | | 0.14 | .07 | .35 |
| MBSR vs. EI ^b^ | 0.17 | 0.18 | .08 | | .34 |  | | -0.22 | | 0.15 | -.13 | .15 |
| Control vs. EI ^c^ | 0.43* | 0.19 | .21 | | .02 |  | | -0.26* | | 0.13 | -.15 | .04 |
| *R^2^* | 0.03 |  |  | | .24 |  | | 0.57* | |  |  | .00 |
| **Gratitude T1** |  |  |  | |  |  | | 0.57* | | 0.06 | .69 | .00 |
| SKY vs. EI ^a^ | 0.16 | 0.24 | .07 | | .52 |  | | 0.00 | | 0.16 | .00 | 1.00 |
| MBSR vs. EI ^b^ | -0.08 | 0.22 | -.04 | | .71 |  | | -0.29 | | 0.16 | -.16 | .07 |
| Control vs. EI ^c^ | 0.22 | 0.22 | .10 | | .32 |  | | -0.27 | | 0.15 | -.15 | .06 |
| *R^2^* | 0.02 |  |  | | .36 |  | | 0.51* | |  |  | .00 |
| **Self-Compassion T1** |  |  |  | |  |  | | 0.47* | | 0.08 | .52 | .00 |
| SKY vs. EI ^a^ | -0.05 | 0.19 | -.03 | | .78 |  | | 0.10 | | 0.19 | .06 | .61 |
| MBSR vs. EI ^b^ | -0.09 | 0.17 | -.05 | | .62 |  | | -0.13 | | 0.18 | -.09 | .47 |
| Control vs. EI ^c^ | 0.20 | 0.18 | .11 | | .25 |  | | -0.25 | | 0.16 | -.16 | .11 |
| *R^2^* | 0.02 |  |  | | .28 |  | | 0.29* | |  |  | .00 |
| **Mindfulness T1** |  |  |  | |  |  | | 0.58* | | 0.07 | .62 | .00 |
| SKY vs. EI ^a^ | 0.24* | 0.11 | .19 | | .04 |  | | -0.05 | | 0.11 | -.04 | .65 |
| MBSR vs. EI ^b^ | 0.16 | 0.10 | .13 | | .13 |  | | -0.17 | | 0.11 | -.15 | .13 |
| Control vs. EI ^c^ | 0.33* | 0.10 | .28 | | .00 |  | | -0.36* | | 0.10 | -.32 | .00 |
| *R^2^* | 0.05 |  |  | | .09 |  | | 0.40* | |  |  | .00 |
| **Adaptive Coping T1** |  |  |  | |  |  | | 0.55* | | 0.07 | .53 | .00 |
| SKY vs. EI ^a^ | -0.04 | 0.13 | -.03 | | .77 |  | | 0.01 | | 0.13 | .01 | .91 |
| MBSR vs. EI ^b^ | -0.02 | 0.12 | -.02 | | .83 |  | | -0.04 | | 0.12 | -.03 | .75 |
| Control vs. EI ^c^ | -0.02 | 0.12 | -.01 | | .90 |  | | -0.25* | | 0.11 | -.20 | .02 |
| *R^2^* | 0.00 |  |  | | .88 |  | | 0.32* | |  |  | .00 |
| **Negative affect T1** |  |  |  |  | | |  | | 0.38* | 0.09 | .42 | .00 |
| SKY vs. EI ^a^ | -0.03 | 0.16 | -.02 | .87 | | |  | | -0.02 | 0.16 | -.01 | .89 |
| MBSR vs. EI ^b^ | -0.06 | 0.15 | -.04 | .67 | | |  | | 0.13 | 0.18 | .10 | .45 |
| Control vs. EI ^c^ | -0.28 | 0.16 | -.18 | .07 | | |  | | 0.26 | 0.15 | .19 | .07 |
| *R^2^* | 0.03 |  |  | .25 | | |  | | 0.19* |  |  | .01 |
| **Maladaptive Coping T1** |  |  |  |  | | |  | | 0.47* | 0.10 | .50 | .00 |
| SKY vs. EI ^a^ | -0.06 | 0.11 | -.05 | .59 | | |  | | 0.02 | 0.11 | .02 | .87 |
| MBSR vs. EI ^b^ | -0.05 | 0.10 | -.05 | .61 | | |  | | 0.09 | 0.11 | .10 | .42 |
| Control vs. EI ^c^ | -0.20* | 0.10 | -.20 | .04 | | |  | | 0.20 | 0.11 | .22 | .08 |
| *R^2^* | 0.03 |  |  | .21 | | |  | | 0.26* |  |  | .00 |

*Notes*. * : *p(two-tailed)* ≤ .05; ^a^ coded 1 = SKY treatment group, 0 = all other groups; ^b^ coded 1 = MBSR treatment group, 0 = all other groups; ^c^ coded 1 = Control group, 0 = all other groups; This table uses 2-tailed p-values because we had no directed hypotheses for the comparisons among the three intervention groups.

**Table K:** Comparing all groups to the EI intervention: From T1 to T2: Regressions for health well-being outcomes regressed on groups

|  | Time 1 | | | |  | Time 2 | | | |
| --- | --- | --- | --- | --- | --- | --- | --- | --- | --- |
| Predictors | *B* | *SE(B)* | β | *p (2-tailed)* |  | *B* | *SE(B)* | β | *p (2-tailed)* |
| **Physical Health T1** |  |  |  |  |  | 0.68* | 0.05 | .77 | .00 |
| SKY vs. EI ^a^ | -0.29 | 0.22 | -.12 | .17 |  | -0.10 | 0.18 | -.05 | .56 |
| MBSR vs. EI ^b^ | -0.29 | 0.22 | -.13 | .18 |  | -0.02 | 0.18 | -.01 | .89 |
| Control vs. EI ^c^ | -0.74* | 0.22 | -.33 | .00 |  | 0.04 | 0.15 | .02 | .78 |
| *R^2^* | 0.07 |  |  | .06 |  | 0.58* |  |  | .00 |
| **Sleep Problems T1** |  |  |  |  |  | 0.27* | 0.08 | .33 | .00 |
| SKY vs. EI ^a^ | 0.82 | 0.82 | .09 | .32 |  | -0.50 | 0.78 | -.06 | .52 |
| MBSR vs. EI ^b^ | 1.19 | 0.82 | .14 | .15 |  | 0.93 | 0.92 | .13 | .31 |
| Control vs. EI ^c^ | -0.59 | 0.78 | -.07 | .45 |  | 0.72 | 0.78 | .10 | .36 |
| *R^2^* | 0.03 |  |  | .20 |  | 0.14* |  |  | .02 |

*Notes*. * : *p(two-tailed)* ≤ .05; ^a^ coded 1 = SKY treatment group, 0 = all other groups; ^b^ coded 1 = MBSR treatment group, 0 = all other groups; ^c^ coded 1 = Control group, 0 = all other groups; This table uses 2-tailed p-values because we had no directed hypotheses for the comparisons among the three intervention groups.

**Table L:** From T1 to T2: Comparing all groups to the MBSR intervention: Comparing all groups to the EI intervention: Regressions for mental health outcomes regressed on groups

|  | Outcome Time 1 | | | |  | Outcome Time 2 | | | |
| --- | --- | --- | --- | --- | --- | --- | --- | --- | --- |
| Predictors | *B* | *SE(B)* | β | *p (2-tailed)* |  | *B* | *SE(B)* | β | *p (2-tailed)* |
| **Burnout T1** |  |  |  |  |  | 0.18* | 0.09 | .18 | .05 |
| SKY vs. MBSR ^a^ | -0.01 | 0.14 | -.01 | .95 |  | -0.09 | 0.17 | -.05 | .63 |
| EI vs. MBSR ^b^ | 0.19 | 0.17 | .10 | .25 |  | 0.04 | 0.21 | .02 | .86 |
| Control vs. MBSR ^c^ | -0.33* | 0.14 | -.20 | .02 |  | 0.32* | 0.17 | .20 | .05 |
| *R^2^* | 0.06 |  |  | .08 |  | 0.06 |  |  | .16 |
| **Stress T1** |  |  |  |  |  | 0.36* | 0.09 | .35 | .00 |
| SKY vs. MBSR ^a^ | 0.02 | 0.13 | .01 | .89 |  | -0.26 | 0.14 | -.18 | .07 |
| EI vs. MBSR ^b^ | 0.09 | 0.13 | .06 | .46 |  | 0.01 | 0.19 | .01 | .95 |
| Control vs. MBSR ^c^ | -0.28* | 0.11 | -.21 | .01 |  | 0.21 | 0.14 | .15 | .13 |
| *R^2^* | 0.06 |  |  | .08 |  | 0.17* |  |  | .01 |
| **Distress T1** |  |  |  |  |  | 0.34* | 0.07 | .41 | .00 |
| SKY vs. MBSR ^a^ | 0.09 | 0.16 | .05 | .58 |  | -0.08 | 0.13 | -.05 | .55 |
| EI vs. MBSR ^b^ | 0.12 | 0.17 | .06 | .49 |  | 0.08 | 0.19 | .05 | .66 |
| Control vs. MBSR ^c^ | -0.09 | 0.14 | -.05 | .54 |  | 0.16 | 0.14 | .11 | .25 |
| *R^2^* | 0.01 |  |  | .51 |  | 0.18* |  |  | .00 |
| **Depression T1** |  |  |  |  |  | 0.47* | 0.08 | .48 | .00 |
| SKY vs. MBSR ^a^ | -0.14 | 0.15 | -.08 | .35 |  | -0.37* | 0.17 | -.21 | .03 |
| EI vs. MBSR ^b^ | -0.13 | 0.16 | -.07 | .42 |  | -0.05 | 0.20 | -.03 | .78 |
| Control vs. MBSR ^c^ | -0.43* | 0.15 | -.25 | .00 |  | 0.13 | 0.14 | .08 | .34 |
| *R^2^* | 0.05 |  |  | .14 |  | 0.27* |  |  | .00 |
| **Anxiety T1** |  |  |  |  |  | 0.51* | 0.06 | .63 | .00 |
| SKY vs. MBSR ^a^ | 0.06 | 0.13 | .04 | .63 |  | -0.01 | 0.09 | -.01 | .89 |
| EI vs. MBSR ^b^ | -0.03 | 0.13 | -.02 | .85 |  | -0.04 | 0.10 | -.03 | .72 |
| Control vs. MBSR ^c^ | -0.14 | 0.11 | -.10 | .20 |  | 0.12 | 0.09 | .11 | .19 |
| *R^2^* | 0.02 |  |  | .38 |  | 0.39* |  |  | .00 |
| **Mental Health T1** |  |  |  |  |  | 0.63* | 0.06 | .64 | .00 |
| SKY vs. MBSR ^a^ | -0.18 | 0.21 | -.07 | .41 |  | -0.78* | 0.23 | -.29 | .00 |
| EI vs. MBSR ^b^ | 0.13 | 0.24 | .05 | .58 |  | -0.59* | 0.24 | -.21 | .01 |
| Control vs. MBSR ^c^ | -0.59* | 0.21 | -.23 | .00 |  | -0.12 | 0.21 | -.05 | .57 |
| *R^2^* | 0.06 |  |  | .09 |  | 0.47* |  |  | .00 |

*Notes*. * : *p(two-tailed)* ≤ .05; ^a^ coded 1 = SKY treatment group, 0 = all other groups; ^b^ coded 1 = EI treatment group, 0 = all other groups; ^c^ coded 1 = Control group, 0 = all other groups; This table uses 2-tailed p-values because we had no directed hypotheses for the comparisons among the three intervention groups.

**Table M:** Comparing all groups to the MBSR intervention: From T1 to T2: Regressions for well-being outcomes regressed on groups

|  | Outcome Time 1 | | | |  | Outcome Time 2 | | | |
| --- | --- | --- | --- | --- | --- | --- | --- | --- | --- |
| Predictors | *B* | *SE(B)* | β | *p (2-tailed)* |  | *B* | *SE(B)* | β | *p (2-tailed)* |
| **Life Satisfaction T1** |  |  |  |  |  | 0.57* | 0.06 | .71 | .00 |
| SKY vs. MBSR ^a^ | -0.10 | 0.24 | -.03 | .68 |  | 0.24 | 0.20 | .10 | .24 |
| EI vs. MBSR ^b^ | -0.01 | 0.28 | .00 | .98 |  | 0.26 | 0.19 | .10 | .18 |
| Control vs. MBSR ^c^ | 0.60* | 0.21 | .22 | .01 |  | -0.12 | 0.16 | -.05 | .47 |
| *R^2^* | 0.06 |  |  | .06 |  | 0.48* |  |  | .00 |
| **Ryff Autonomy T1** |  |  |  |  |  | 0.59 | 0.05 | .69 | .00 |
| SKY vs. MBSR ^a^ | 0.10 | 0.28 | .03 | .72 |  | 0.00 | 0.18 | .00 | .99 |
| EI vs. MBSR ^b^ | 0.08 | 0.30 | .02 | .80 |  | -0.33 | 0.25 | -.11 | .19 |
| Control vs. MBSR ^c^ | 0.05 | 0.24 | .02 | .85 |  | -0.38 | 0.17 | -.15 | .03 |
| *R^2^* | 0.00 |  |  | .85 |  | 0.50 |  |  | .00 |
| **Ryff Environment T1** |  |  |  |  |  | 0.49 | 0.08 | .51 | .00 |
| SKY vs. MBSR ^a^ | 0.09 | 0.23 | .03 | .69 |  | 0.48 | 0.25 | .19 | .06 |
| EI vs. MBSR ^b^ | -0.17 | 0.24 | -.06 | .48 |  | 0.21 | 0.27 | .08 | .44 |
| Control vs. MBSR ^c^ | 0.60 | 0.20 | .24 | .00 |  | -0.20 | 0.21 | -.08 | .33 |
| *R^2^* | 0.07 |  |  | .05 |  | 0.28 |  |  | .00 |
| **Ryff Growth T1** |  |  |  |  |  | 0.43 | 0.07 | .50 | .00 |
| SKY vs. MBSR ^a^ | -0.01 | 0.14 | -.01 | .93 |  | 0.12 | 0.14 | .08 | .41 |
| EI vs. MBSR ^b^ | -0.08 | 0.16 | -.04 | .63 |  | 0.18 | 0.12 | .11 | .14 |
| Control vs. MBSR ^c^ | 0.01 | 0.13 | .00 | .96 |  | -0.11 | 0.12 | -.08 | .37 |
| *R^2^* | 0.00 |  |  | .79 |  | 0.27 |  |  | .00 |
| **Ryff Relations T1** |  |  |  |  |  | 0.64 | 0.05 | .74 | .00 |
| SKY vs. MBSR ^a^ | 0.34 | 0.28 | .11 | .23 |  | 0.34 | 0.22 | .12 | .12 |
| EI vs. MBSR ^b^ | 0.04 | 0.30 | .01 | .89 |  | 0.06 | 0.21 | .02 | .77 |
| Control vs. MBSR ^c^ | 0.45 | 0.24 | .15 | .06 |  | -0.09 | 0.18 | -.03 | .64 |
| *R^2^* | 0.02 |  |  | .29 |  | 0.57 |  |  | .00 |
| **Ryff Purpose T1** |  |  |  |  |  | 0.57 | 0.08 | .57 | .00 |
| SKY vs. MBSR ^a^ | 0.04 | 0.20 | .02 | .86 |  | 0.18 | 0.21 | .08 | .41 |
| EI vs. MBSR ^b^ | -0.29 | 0.23 | -.12 | .21 |  | 0.25 | 0.24 | .10 | .30 |
| Control vs. MBSR ^c^ | 0.08 | 0.17 | .04 | .64 |  | 0.12 | 0.19 | .06 | .51 |
| *R^2^* | 0.02 |  |  | .39 |  | 0.33 |  |  | .00 |
| **Ryff Self-Acceptance** |  |  |  |  |  | 0.44 | 0.05 | .56 | .00 |
| SKY vs. MBSR ^a^ | 0.08 | 0.24 | .03 | .73 |  | 0.29 | 0.19 | .13 | .13 |
| EI vs. MBSR ^b^ | 0.05 | 0.29 | .02 | .88 |  | 0.17 | 0.20 | .07 | .40 |
| Control vs. MBSR ^c^ | 0.44 | 0.21 | .17 | .04 |  | -0.28 | 0.20 | -.13 | .16 |
| *R^2^* | 0.02 |  |  | .24 |  | 0.33 |  |  | .00 |
| **Pleasant Emotions T1** |  |  |  |  |  | 0.56* | 0.07 | .54 | .00 |
| SKY vs. MBSR ^a^ | 0.21 | 0.14 | .12 | .14 |  | 0.10 | 0.17 | .06 | .55 |
| EI vs. MBSR ^b^ | 0.10 | 0.14 | .06 | .47 |  | -0.25 | 0.15 | -.14 | .10 |
| Control vs. MBSR ^c^ | 0.31* | 0.13 | .20 | .02 |  | -0.41* | 0.13 | -.26 | .00 |
| *R^2^* | 0.03 |  |  | .22 |  | 0.35* |  |  | .00 |
| **Connectedness T1** |  |  |  |  |  | 0.65* | 0.06 | .75 | .00 |
| SKY vs. MBSR ^a^ | 0.04 | 0.19 | .02 | .85 |  | 0.35* | 0.14 | .19 | .01 |
| EI vs. MBSR ^b^ | -0.17 | 0.18 | -.07 | .34 |  | 0.22 | 0.15 | .11 | .15 |
| Control vs. MBSR ^c^ | 0.26 | 0.17 | .13 | .13 |  | -0.04 | 0.13 | -.03 | .74 |
| *R^2^* | 0.03 |  |  | .24 |  | 0.57* |  |  | .00 |
| **Gratitude T1** |  |  |  |  |  | 0.57* | 0.06 | .69 | .00 |
| SKY vs. MBSR ^a^ | 0.24 | 0.21 | .10 | .25 |  | 0.29* | 0.15 | .15 | .05 |
| EI vs. MBSR ^b^ | 0.08 | 0.22 | .03 | .71 |  | 0.29 | 0.16 | .14 | .07 |
| Control vs. MBSR ^c^ | 0.30 | 0.17 | .14 | .09 |  | 0.02 | 0.14 | .01 | .89 |
| *R^2^* | 0.02 |  |  | .36 |  | 0.51* |  |  | .00 |
| **Self-Compassion T1** |  |  |  |  |  | 0.47* | 0.08 | .52 | .00 |
| SKY vs. MBSR ^a^ | 0.03 | 0.16 | .02 | .84 |  | 0.23 | 0.17 | .14 | .17 |
| EI vs. MBSR ^b^ | 0.09 | 0.17 | .04 | .62 |  | 0.13 | 0.18 | .07 | .47 |
| Control vs. MBSR ^c^ | 0.29* | 0.14 | .16 | .04 |  | -0.12 | 0.13 | -.07 | .39 |
| *R^2^* | 0.02 |  |  | .28 |  | 0.29* |  |  | .00 |
| **Mindfulness T1** |  |  |  |  |  | 0.58* | 0.07 | .62 | .00 |
| SKY vs. MBSR ^a^ | 0.08 | 0.11 | .07 | .45 |  | 0.12 | 0.11 | .10 | .27 |
| EI vs. MBSR ^b^ | -0.16 | 0.10 | -.12 | .13 |  | 0.17 | 0.11 | .13 | .13 |
| Control vs. MBSR ^c^ | 0.18 | 0.10 | .15 | .08 |  | -0.19* | 0.10 | -.18 | .04 |
| *R^2^* | 0.05 |  |  | .09 |  | 0.40* |  |  | .00 |
| **Adaptive Coping T1** |  |  |  |  |  | 0.55* | 0.07 | .53 | .00 |
| SKY vs. MBSR ^a^ | -0.01 | 0.11 | -.01 | .91 |  | 0.05 | 0.13 | .04 | .69 |
| EI vs. MBSR ^b^ | 0.02 | 0.12 | .02 | .83 |  | 0.04 | 0.12 | .03 | .75 |
| Control vs. MBSR ^c^ | 0.01 | 0.10 | .01 | .92 |  | -0.21* | 0.11 | -.17 | .05 |
| *R^2^* | 0.00 |  |  | .88 |  | 0.32* |  |  | .00 |
| **Negative affect T1** |  |  |  |  |  | 0.38* | 0.09 | .42 | .00 |
| SKY vs. MBSR ^a^ | 0.04 | 0.14 | .02 | .79 |  | -0.15 | 0.15 | -.10 | .31 |
| EI vs. MBSR ^b^ | 0.06 | 0.15 | .04 | .67 |  | -0.13 | 0.18 | -.08 | .45 |
| Control vs. MBSR ^c^ | -0.22 | 0.13 | -.14 | .08 |  | 0.13 | 0.14 | .10 | .35 |
| *R^2^* | 0.03 |  |  | .25 |  | 0.19* |  |  | .01 |
| **Maladaptive Coping T1** |  |  |  |  |  | 0.47* | 0.10 | .50 | .00 |
| SKY vs. MBSR ^a^ | -0.01 | 0.09 | -.01 | .94 |  | -0.07 | 0.07 | -.07 | .30 |
| EI vs. MBSR ^b^ | 0.05 | 0.10 | .04 | .61 |  | -0.09 | 0.11 | -.09 | .42 |
| Control vs. MBSR ^c^ | -0.15 | 0.08 | -.15 | .06 |  | 0.11 | 0.08 | .12 | .17 |
| *R^2^* | 0.03 |  |  | .21 |  | 0.26* |  |  | .00 |

*Notes*. * : *p(two-tailed)* ≤ .05; ^a^ coded 1 = SKY treatment group, 0 = all other groups; ^b^ coded 1 = EI treatment group, 0 = all other groups; ^c^ coded 1 = Control group, 0 = all other groups; This table uses 2-tailed p-values because we had no directed hypotheses for the comparisons among the three intervention groups.

**Table N:** Comparing all groups to the MBSR intervention: From T1 to T2: Regressions for health well-being outcomes regressed on groups

|  | Time 1 | | | |  | Time 2 | | | |
| --- | --- | --- | --- | --- | --- | --- | --- | --- | --- |
| Predictors | *B* | *SE(B)* | β | *p (2-tailed)* |  | *B* | *SE(B)* | β | *p (2-tailed)* |
| **Physical Health T1** |  |  |  |  |  | 0.68* | 0.05 | .77 | .00 |
| SKY vs. MBSR ^a^ | 0.00 | 0.20 | .00 | 1.00 |  | -0.08 | 0.16 | -.04 | .62 |
| EI vs. MBSR ^b^ | 0.29 | 0.22 | .11 | .18 |  | 0.02 | 0.18 | .01 | .89 |
| Control vs. MBSR ^c^ | -0.45* | 0.20 | -.20 | .02 |  | 0.07 | 0.13 | .03 | .62 |
| *R^2^* | 0.07 |  |  | .06 |  | 0.58* |  |  | .00 |
| **Sleep Problems T1** |  |  |  |  |  | 0.27* | 0.08 | .33 | .00 |
| SKY vs. MBSR ^a^ | -0.36 | 0.82 | -.04 | .66 |  | -1.43 | 0.75 | -.19 | .06 |
| EI vs. MBSR ^b^ | -1.19 | 0.82 | -.12 | .15 |  | -0.93 | 0.92 | -.11 | .31 |
| Control vs. MBSR ^c^ | -1.78* | 0.78 | -.20 | .02 |  | -0.21 | 0.80 | -.03 | .79 |
| *R^2^* | 0.03 |  |  | .20 |  | 0.14* |  |  | .02 |

*Notes*. * : *p(two-tailed)* ≤ .05; ^a^ coded 1 = SKY treatment group, 0 = all other groups; ^b^ coded 1 = EI treatment group, 0 = all other groups; ^c^ coded 1 = Control group, 0 = all other groups; This table uses 2-tailed p-values because we had no directed hypotheses for the comparisons among the three intervention groups.

**Figure 1.** Results from acceptability items

[Confidence M(SD) = SKY: 3.14 (.88); MBSR: 2.79 (.84); EI: .43 (.68)]
